# A divide-and-conquer approach for genomic prediction in rubber tree using machine learning

**DOI:** 10.1038/s41598-022-20416-z

**Published:** 2022-10-26

**Authors:** Alexandre Hild Aono, Felipe Roberto Francisco, Livia Moura Souza, Paulo de Souza Gonçalves, Erivaldo J. Scaloppi Junior, Vincent Le Guen, Roberto Fritsche-Neto, Gregor Gorjanc, Marcos Gonçalves Quiles, Anete Pereira de Souza

**Affiliations:** 1 Molecular Biology and Genetic Engineering Center (CBMEG), University of Campinas (UNICAMP), Campinas, SP Brazil; 2 The Roslin Institute and Royal (Dick) School of Veterinary Studies, The University of Edinburgh, Midlothian, UK; 3 São Francisco University (USF), Itatiba, Brazil; 4 Center of Rubber Tree and Agroforestry Systems, Agronomic Institute (IAC), Votuporanga, Brazil; 5 Centre de Coopération Internationale en Recherche Agronomique pour le Développement (CIRAD), UMR AGAP, 34398 Montpellier, France; 6 AGAP, CIRAD, INRAE, Institut Agro, Univ Montpellier, Montpellier, France; 7 Genetics Department, Luiz de Queiroz College of Agriculture (ESALQ), University of São Paulo (USP), Piracicaba, SP Brazil; 8 Instituto de Ciência e Tecnologia (ICT), Universidade Federal de São Paulo (UNIFESP), São José dos Campos, SP Brazil; 9 Department of Plant Biology, Institute of Biology (IB), University of Campinas (UNICAMP), Campinas, SP Brazil

**Keywords:** Computational biology and bioinformatics, Genetics, Plant sciences

## Abstract

Rubber tree (*Hevea brasiliensis*) is the main feedstock for commercial rubber; however, its long vegetative cycle has hindered the development of more productive varieties via breeding programs. With the availability of *H. brasiliensis* genomic data, several linkage maps with associated quantitative trait loci have been constructed and suggested as a tool for marker-assisted selection. Nonetheless, novel genomic strategies are still needed, and genomic selection (GS) may facilitate rubber tree breeding programs aimed at reducing the required cycles for performance assessment. Even though such a methodology has already been shown to be a promising tool for rubber tree breeding, increased model predictive capabilities and practical application are still needed. Here, we developed a novel machine learning-based approach for predicting rubber tree stem circumference based on molecular markers. Through a divide-and-conquer strategy, we propose a neural network prediction system with two stages: (1) subpopulation prediction and (2) phenotype estimation. This approach yielded higher accuracies than traditional statistical models in a single-environment scenario. By delivering large accuracy improvements, our methodology represents a powerful tool for use in *Hevea* GS strategies. Therefore, the incorporation of machine learning techniques into rubber tree GS represents an opportunity to build more robust models and optimize *Hevea* breeding programs.

## Introduction

Rubber tree (*Hevea brasiliensis*) has an elevated importance in the global economy, being almost the only feedstock for commercial rubber^1,2^. Considering the long perennial vegetative cycle of *Hevea*, breeding programs aim to improve its yield production in order to reach the rapidly increasing rubber demand^1–3^. Therefore, genomic approaches are needed in rubber tree breeding, especially considering its recent domestication history^4^. *H. brasiliensis* is a diploid species ($2n=36$) with an elevated occurrence of duplicated regions in its genome ($\sim 70\%$)^5–7^, and this complex genomic organization has hindered the development of genomic strategies for breeding. However, with the improvement of next-generation sequencing (NGS) technologies and the consequent reduction in genotyping costs, data generation has become more efficient, providing more genomic resources in less time and with lower associated costs^8^. This greater availability of data improved precision in selection with higher genetic gains in various crops^8,9^ and, in rubber tree, could complement traditional approaches based on only phenotypic and pedigree information^8,10^.

Various rubber tree genomic resources have become available in recent decades, such as a large set of different molecular markers^11–14^, draft genomes^5,6^, and, more recently, a chromosome-level assembled genome^7^. These data have already allowed the construction of saturated linkage maps with associated quantitative trait loci (QTLs), which were proposed as a tool for marker-assisted selection (MAS)^15^. Although QTLs for several traits have been identified in rubber tree^4,15–20^, the amount of phenotypic variance explained by these identified QTLs is usually small^19^ because of the highly complex genetic architectures associated with growth and rubber production traits. The configuration of these phenotypes is controlled by many genes with small effects^21^, and weak QTLs may not be identified using existing methodologies^2,22^, which prevents the identification of interindividual differences^23^. Together with the environmental and genetic background restrictions of QTLs^24^, these features limit the application of *Hevea* QTLs for MAS^14^. Consequently, novel genomic strategies that can assist in rubber tree breeding programs are needed, especially considering the time required to evaluate these phenotypes, the elevated costs, and the low female fertility in *H. brasiliensis*^2,15,25^.

Aimed at solving such difficulties in many crops, genomic selection (GS) has arisen as a promising methodology for considerably reducing the required breeding cycle^26^. GS has shown better performance than MAS^27,28^, mainly because of its associated genetic gains^29^ and reduced costs over a long time period^30^. This strategy enables the selection of plants based on their estimated performance obtained with a large dataset of molecular markers^8,31^, reducing breeding time by avoiding the need to evaluate a considerable number of phenotypes over different years^24^. Using known phenotypic and genotypic information from a training population^32^, it is possible to create a predictive model that can be used to predict the breeding values of a testing population using only genotypic data^8^. This modeling is generally based on a mixed-effect regression method^33^ and has already been demonstrated to be promising for several crops^34–38^. In rubber tree^25^, and^2^ assessed the potential of GS for predicting stem circumference (SC) and rubber production (RP), respectively, simulating breeding schemes through cross-validation (CV) techniques.

There are several CV approaches for simulating a real application of GS in a plant breeding program. These methods take into account the population structure in the dataset and the appropriateness of applying the developed predictive model to a set of plants. There are basically three CV schemes in GS: (1) predicting traits in an untested environment using previously tested lines (CV0)^8^, (2) predicting new lines’ traits that were not evaluated in any environment (CV1)^39^, and (3) predicting traits that were evaluated in some environments but not in others (CV2)^40^. These three scenarios were already evaluated in rubber tree^2^ assessed the potential of GS in a within-family context using CV0 and CV1 methods, and^25^ tested three different populations with CV1 and CV2. These initiatives represent the first attempts to use GS on rubber tree data, but with low associated predictive capabilities for some of the created CV schemes, mostly when prediction is performed with genotypes that have not already been tested.

Different approaches have been used in GS to create predictive models, including parametric and nonparametric methods^24,26,41–45^. Significant differences in predictive capabilities have not been demonstrated when changing the predictive approach^31,46,47^; thus, linking genotypes and phenotypes remains a great challenge^23,48^, especially for plant species with high genomic complexity. In this context, more robust techniques for estimating these models with higher prediction capabilities are needed to expand the practical implementation of GS in rubber tree. Nonlinear techniques have already shown improved performance in representing complex traits with nonadditive effects^9,49–51^, and, in this context, machine learning (ML) strategies have emerged as a promising set of tools for complementing these statistical nonlinear methods.

The objective of this work was to develop a genomic prediction approach for rubber tree data. Considering that ML methods have not been proven to have better performance than statistical methodologies for GS^23,52^, we evaluated their efficiency in rubber tree, also suggesting a novel approach for constructing a predictive system with neural networks based on two-stage prediction: (1) subpopulation prediction and (2) phenotype estimation. Such a divisive approach was created considering a common paradigm in Computer Science: divide and conquer. For datasets with a clear subpopulation structure, such as rubber tree, the proposed approach represents a promising alternative for the development of predictive models.

## Material and methods

### Plant material and phenotypic characterization

The data used in this work were obtained with different experiments in two previous studies. The plant material and permissions for collecting rubber tree employed in the present study are in compilance with institutional, national, and international guidelines and legislation. Therefore, our analyses were conducted by separating the methodologies and considering two datasets: experimental group 1 (EG1) and experimental group 2 (EG2). EG1 includes 408 samples of three $F_1$ segregant populations obtained with crosses between (Pop1) GT1 and PB235 (30 genotypes)^25^, (Pop2) GT1 and RRIM701 (127 genotypes)^25,53^, and (Pop3) PR255 and PB217 (251 genotypes)^4,19,25^. EG2 is based on an $F_1$ cross between RRIM600 and PB260 (330 samples)^2^.

The parents of the crosses used are important clones for rubber tree breeding programs. PR255, PB235, PB260, and RRIM600 have high yield, and PB217 has considerable potential for long-term yield performance due to its slow growth process^2,25^. PR255 and RRIM701 have good growth, and RRIM701 also presents an increased SC after initial tapping^54^. The latex production is stable in PR255 and medium in RRIM600. Stable or medium latex production represents a good adaptation to several environments, as observed in GT1, a clone tolerant to wind and cold. Additionally, PB260 presents high female fertility^55^, and PB235 is susceptible to tapping panel dryness^56^.

In EG1 and EG2, we analyzed the SC trait. In EG1, Pop3 was planted in 2006 in a randomized block design in Itiquira, Mato Grosso State, Brazil, 17$^{\circ }$24$'$ 03$''$ S and 54$^{\circ }$44$'$ 53$''$ W^4,19,25^. Each individual was represented by four grafted trees in each plot and four replications. Pop1 and Pop2 were planted in 2012 at the Center of Rubber Tree and Agroforestry Systems/Agronomic Institute (IAC - Brazil), 20$^{\circ }$25$'$ 00$''$ S and 49$^{\circ }$59$'$ 00$''$ W, following an augmented block design, with four blocks containing two clones per plot spaced 4 m apart for each trial, which was repeated four times^25,53^.

Even though EG2 corresponds to only one cross, this population was planted following an almost complete block design at two different sites^2^, which for convenience we named site 1 (S1) and site 2 (S2). In S1, 189 clones were planted in 2012 in Société des Caoutchoucs de Grand-Béréby (SOGB—Ivory Coast), 4° 40′ 54″ N and 7° 06′ 05″ W. In S2, 143 clones were planted in 2013 in Société Africaine de Plantations d’Hévéas (SAPH - Ivory Coast), 5° 19′ 47.79″ N and 4° 36′ 39.74″ W. This cross consisted of six blocks with randomized trees spaced 2.5 m apart and a mean number of ramets per clone of 11 for S1 (ranging between 7 and 17) and 13 for S2 (ranging between 5 and 20).

SC measurements of Pop3 in EG1 were obtained in four years (from 2007 to 2010) and those of Pop1 and Pop2 were obtained from 2013 to 2016, considering that growth traits are usually measured only during the first 6 years^25,57^. According to the water distribution of the experiments installed, EG1 phenotypes were measured to supply information considering low-water (LW) and well-watered (WW) conditions; thus, Pop3 was evaluated in October 2007–2010 (LW) and in April 2008–2010 (WW), and Pop1 and Pop2 were evaluated in June 2013, December 2013, May 2014, November 2014, and June 2015–2016. SCs were measured for individual trees at 50 cm above ground level. For both phenotypes, the average per plot was calculated. SC in EG2 was measured at 1 m above ground level before tapping for 3 months every two days except on Sundays (with the beginning at 32 months after planting in S1 and 38 months after planting in S2).

### Phenotypic data analysis

All phenotypic analyses were performed using R statistical software^58^. EG1 and EG2 traits were analyzed with the following steps: (1) data distribution evaluation; (2) standardized normalization with the R package bestNormalize^59^; (3) mixed-effect model creation and residual appropriateness verification through quantile-quantile (Q-Q) plots using the breedR package^60^; (4) estimation of best linear unbiased predictions (BLUPs) based on the models created; (5) hierarchical clustering on BLUP values using a complete hierarchical clustering approach based on Euclidean distances and dendrogram visualization with the ggtree R package^61^; and (6) identification of phenotypic groups using the clustering approach of (5), with cluster numbers ranging between 2 and 5, and several clustering indexes implemented in the NbClust R package^62^.

In EG1, we employed the following statistical mixed-effect model:

1 $$\begin{aligned} Y_{ijk}=\mu +L_k+B_{jk}+W+G_{ik}+e_{ijk} \end{aligned}$$

where $Y_{ijk}$ corresponds to the phenotype of the *i*th genotype in the *j*th block and *k*th location. The phenotypic mean is represented by $\mu$, and the fixed effects represent the contribution of the *k*th location ($L_k$), the *j*th block at the *k*th location ($B_{jk}$), and the watering condition of the measurement (*W*). The genotype *G* and the residual error *e* (nongenetic effects) represent the random effects.

EG2 SC phenotypes were modeled for each site (S1 and S2) according to the following statistical model:

2 $$\begin{aligned} Y_{ijkr}=\mu +B_j+L_{kj}+R_{rkj}+G_{ij}+e_{ijkr} \end{aligned}$$

where $Y_{ijkr}$ corresponds to the phenotype of the *i*th genotype positioned in the *r*th rank of the *k*th line in the *j*th block. The phenotypic mean is represented by $\mu$, and the fixed effects represent the contribution of the *j*th block ($B_j$), the *k*th line of the *j*th block ($L_{kj}$), and the *r*th rank of the *k*th line in the *j*th block ($R_{rkj}$). The genotype *G* and the residual error *e* (nongenetic effects) represent the random effects. Broad-sense heritability ($H^2$) was estimated as $H^2=\sigma ^2_g / \sigma ^2_p$, with $\sigma ^2_g$ and $\sigma ^2_p$ representing the genetic and phenotypic variances, respectively.

### Genotyping process

DNA extraction from EG1 was described by^19,53^, and the genotyping process was performed using a genotyping-by-sequencing (GBS) protocol^63^ with *EcoT22I* restriction enzyme followed by Illumina sequencing using the HiSeq platform for Pop3 and the GAIIx platform for Pop1 and Pop2^25^. EG1 genotype data analysis was performed as described by^25^. In summary, raw sequencing reads were processed using the TASSEL 5.0 pipeline^64^, with a minimum count of 6 reads for creating a tag. The tag mapping process was performed using Bowtie2 v.2.1^65^ with the *very sensitive* algorithm and *H. brasiliensis* reference genome^7^. Single nucleotide polymorphisms (SNPs) were called with the TASSEL algorithm, and only biallelic SNPs were retained using VCFtools^66^. These markers were filtered using the R package snpReady^67^ with a maximum of 20% missing data for a SNP and 50% in an individual and a minimum allele frequency (MAF) of 5%. Missing data were imputed using the k-nearest neighbors^68^ algorithm implemented in the snpReady package.

EG2 samples were genotyped with simple sequence repeat (SSR) markers, following the protocol for DNA extraction and genotyping described by^69^. EG2 genotype data analysis was performed as described by^2^. In summary, a total of 332 SSRs were used for S1^20^ and 296 for S2^2^. Missing data were imputed using BEAGLE 3.3.2^70^ with 25 iterations of the phasing algorithm and 20 haplotype pairs to sample for each individual in an iteration. For evaluating the genotypic profile of individuals in EG1 and EG2, we performed principal component analyses (PCAs) in R statistical software^58^ with the ggplot2 package^71^. Additionally, for evaluating the overall correspondences between genotypic and phenotypic data, we colored the PCA scatter plots with the BLUPs estimated for SC trait, as performed by^72^.

### Statistical models for genomic prediction

We employed two different strategies for creating traditional genomic prediction models: Bayesian ridge regression (BRR)^73^ and a single-environment, main genotypic effect model with a Gaussian kernel (SM-GK)^74^. BRR and SM-GK models were implemented in the BGLR^75^ and BGGE^76^ R packages, respectively. Considering the genotype matrix with *n* individuals and *p* markers, BRR models were implemented considering the following:

3 $$\begin{aligned} y=1\mu +Z\gamma +e \end{aligned}$$

where *y* represents the BLUP values calculated based on the established mixed-effect models for phenotypic data analyses, $\mu$ the overall mean, *Z* the genotype matrix, *e* the residuals, and $\gamma$ the vector of marker effects. In SM-GK, *Z* is the incidence matrix of genetic effects, and $\gamma$ is the vector of genetic effects with variance estimated through a Gaussian kernel calculated using the snpReady R package.

### Genomic prediction via machine learning

For genomic prediction via ML, we selected the following algorithms considering a regression task: (a) AdaBoost^77^, (b) multilayer perceptron (MLP) neural networks^78^, (c) random forests^79^, and (d) support vector machine (SVM)^80^. To create these models, we used Python v.3 programming language together with the library scikit-learn v.0.19.0^81^. We also tested a combination of feature selection (FS) techniques for increasing the predictive accuracies^82^, using a combination of three different methods: (i) L1-based FS through an SVM model^80^, (ii) univariate FS with Pearson correlations (and ANOVA for discrete variables) (p-value of 0.05), and (iii) gradient tree boosting^83^. Such a strategy is based on marker subset selection, separating the markers identified by all of these methods together (intersection of the 3 approaches, named Inter3) or by at least two of them simultaneously (Inter2), and using such subsets for prediction.

To understand the subset selection, we performed functional annotation of the genomic regions underlying these markers selected through FS considering a 10,000 base-pair (bp) window for the up- and downstream regions. Using BLASTn software^84^ (minimum e-value of 1e-6), these sequences were aligned against coding DNA sequences (CDSs) from the *Malpighiales* clade (*Linum usitatissimum* v1.0, *Manihot esculenta* v8.1, *Populus deltoides* WV94 v2.1, *Populus trichocarpa* v4.1, *Ricinus communis* v0.1, and *Salix purpurea* v5.1) of the Phytozome v.13 database^85^. On the basis of significant correspondence, Gene Ontology (GO) terms^86^ were retrieved.

### Multilayer perceptron neural network

As the final approach for genomic prediction in EG1, we proposed the creation of neural networks with novel architectures for each of the biparental populations, using the Keras Python v.3 library for this task^87^. We employed MLP networks, which have an architecture based on multiple layers and feedforward signal propagation^88^.

For all the predictive tasks, we considered an MLP structure with two hidden layers (HLs) and used the mean absolute error (MAE) as the error function for training and defining the architecture of the networks. Additionally, 200 epochs were considered (batch size of 16). The training process of the networks was performed using the backpropagation strategy together with the Adam optimization algorithm^89^, which aims to minimize the MAE by updating the synaptic weights using a gradient-based strategy that combines heuristics from a momentum term and RMSProp^90^. The update process is based on a change of $\Delta w_{ij}$ for each connection, considering the individual influence of a weight $w_{ij}$ on the MAE value obtained with the gradient descent $g_t$ in the iteration *t* calculated with $\partial MAE/\partial w_{ij}$ and used in the equation

4 $$\begin{aligned} \Delta w_{ij} = g_t \times \eta \frac{v_t}{\sqrt{s_t+\epsilon }} \end{aligned}$$

where $\eta$ is the learning rate representing the amount of change in the process of training, $v_t$ is the exponential average of gradients along the weights $w_i$ of layer *i*, and $s_t$ is the exponential average of squares of gradients along $w_i$. The Adam optimizer employs two other hyperparameters for the optimization process ($\beta _1$ and $\beta _2$), which are used for the calculation of $v_t$ ($v_t=\beta _1\times v_{t-1}-(1-\beta _1)\times g_t$) and $s_t$ ($s_t=\beta _2\times s_{t-1}-(1-\beta _2)\times g_t^2$). We used $\beta _1=0.9$ and $\beta _2=0.999$^89^. We tested the following configurations for the MLP hyperparameters: (a) number of neurons in the first HL, varying from 1 to $\sqrt{(q+2)m}+2\sqrt{m/(q+2)}$ (*m* individuals and *q* output neurons in the output layer); (b) number of neurons in the second HL, varying from 1 to $q\sqrt{m/(q+2)}$; (c) rectified linear activation (ReLU), sigmoid and hyperbolic tangent activation functions; and (d) learning rates of 0.005, 0.001, and 0.0001. The performed tests for the network definition were based on the upper bounds established by^91,92^.

### Proposed approach and validation strategies

**Figure 1:**
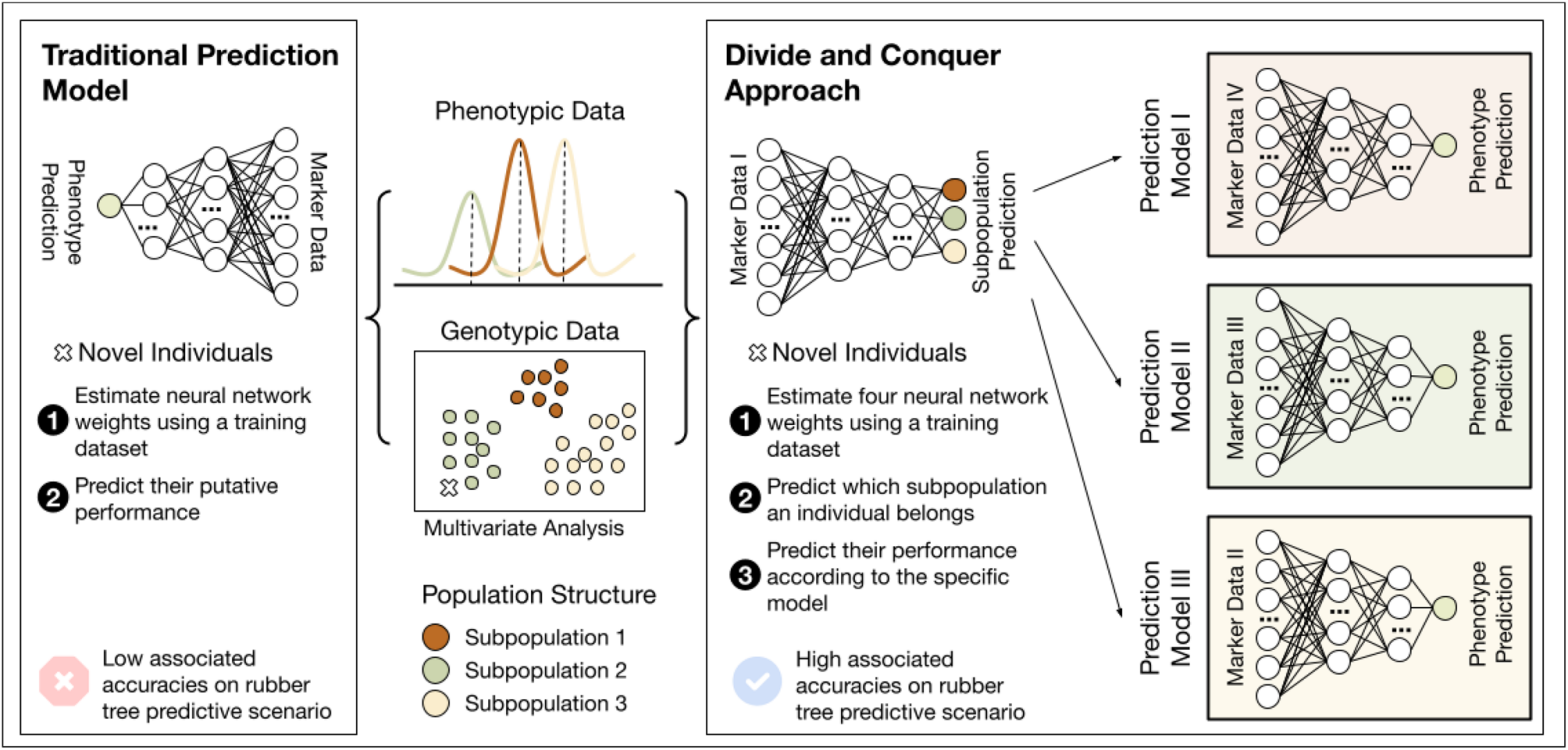
Overview of the approach proposed. Based on a divide-and-conquer strategy with different neural networks combined into a single model (part 1), individuals with unknown phenotypic performance (**a**) are classified into a subpopulation using a specific neural network (part 2) and (**b**) have their phenotypic values estimated through an induced network specific to the subpopulation they belong to (part 3).

Each of the sets of hyperparameters estimated for the MLP networks was used to create a joint and single system for prediction in EG1, which we indicate as part of a divide-and-conquer approach created for genomic prediction (Fig. 1). Considering an individual as part of a dataset subpopulation that has a specific phenotypic distribution, we propose the use of a two-stage prediction process based on the following steps: (1) creating four different neural networks according to different hyperparameter searches and the training data (division step), (2) predicting which subpopulation an unlabeled observation belongs to according to the network induced for this task (prediction 1 and conquer step), and (3) predicting its phenotypic performance based on the network trained specifically for the subpopulation predicted (prediction 2 and final conquer step).

CV1 was the strategy employed for the selection of data for evaluating the models’ performance due to its reduced bias when splitting the dataset and the low prediction accuracies described^25^. We first separated a test dataset using 10% of the genotypes with a stratified holdout strategy implemented in the scikit-learn Python v.3 module^81^. The stratification was performed only in EG1 and was based on the subpopulation structure present in the dataset. For all the models evaluated in this work (statistical and ML based), the same dataset split was considered in every round of CV.

The remaining 90% of the genotypes were used as the development set for defining the networks’ architecture and for evaluating the overall models’ performance through a stratified k-fold approach (k = 4) with 50 repetitions (subpopulation stratification). The predictive accuracy in every CV split was evaluated by comparing the predicted and real BLUPs by measuring (1) the Pearson correlation coefficient (*R*) and (2) the mean absolute percentage error (MAPE). For the subpopulation prediction task, we evaluated the classification accuracy (ratio between the number of correctly predicted data and the total number of predictions). For each trait, we compared the predictive accuracy differences using ANOVA and multiple comparisons by Tukey’s test with the agricolae R package^93^.

For EG1, four different MLP architectures were estimated: (a) subpopulation prediction, (b) BLUP prediction for Pop1, (c) BLUP prediction for Pop2, and (d) BLUP prediction for Pop3. After defining the network hyperparameters with the development set, all of these structures were joined into a single predictive system that was used for the final prediction. In addition to evaluating the predictive performance through the CV scenarios created, we also checked the performance of the model for a leave-one-out (LOO) CV configuration.

## Results

### Phenotypic and genotypic data analyses

The raw phenotypic data were evaluated considering the experimental groups proposed. EG1 (Supplementary Fig. S1) had reduced values compared to those of EG2 (Supplementary Fig. S2) due to the different heights and years of stem measurements. However, for the normalized SC values (Supplementary Figs. S3–S5), such an evident discrepancy was not observed. By modeling the phenotypic measures with the mixed-effect models established and contrasting the raw values with the normalized ones through Q-Q plots, we observed that the residuals obtained with the normalized measurements in EG1 (Supplementary Fig. S6) and EG2 (Supplementary Figs. S7, S8) were more appropriate. Heritabilities ($H^2$) were estimated as 0.55 for EG1, 0.83 for EG2-S1 and 0.93 for EG2-S2, which is in accordance with the findings of^2,25^.

Interestingly, BLUPs from EG1 (Supplementary Fig. S9) and EG2-S1 (Supplementary Fig. S10) presented reduced variability when compared to that of BLUPs estimated for EG2-S2 (Supplementary Fig. S10). This observation is corroborated by the hierarchical clustering analyses performed for these experimental groups. EG1 (Supplementary Fig. S10) and EG2-S1 (Supplementary Fig. S12) could be divided into three phenotypic groups according to the best data partitioning scheme established through NbClust clustering indexes^62^, and EG2-S2 could be arranged into 5 such groups (Supplementary Fig. S13). Therefore, it was expected that for the genomic prediction step, EG2-S2 would represent a more difficult task due to its higher data variability.

SNP calling in EG1 was performed according to the TASSEL pipeline. Of the 363,641 tags produced, approximately 84.78% could be aligned against the *H. brasiliensis* reference genome, which generated 107,466 SNPs. These markers were filtered separately for each population using the parameters established, and then these separated datasets were combined through intersection comparisons, yielding a final dataset of 7414 high-quality SNP markers. For EG2 predictions, 332 and 296 SSR markers were used for EG2-S1 and EG2-S2, respectively.

Using these datasets, we performed PCAs for EG1 (Supplementary Fig. S14) and EG2 (Supplementary Fig. S15). In the figures, the colors of the genotypes correspond to their BLUP values, and their shapes correspond to population structure in EG1 and site in EG2. As expected, for the SC trait, there were no clear associations between markers and BLUPs, underlining the challenge of creating genomic prediction models. Additionally, the subpopulation structure in EG1 was evident.

### Genomic prediction

From the BLUP and marker datasets, we fit genomic prediction models using the traditional statistical approaches (BRR and SM-GK) and the ML algorithms (AdaBoost, MLP, RF, and SVM) selected. For EG1 (Supplementary Fig. S16), EG2-S1 (Supplementary Fig. S17) and EG2-S2 (Supplementary Fig. S18), no substantial changes were observed when changing the prediction approach. After applying Tukey’s multiple comparisons test, we found equivalent performance values for SVM, SM-GK and BRR for all the experimental groups. The worst performance was observed for MLP, however, considering the default architectures employed in scikit-learn^81^.

Additionally, we also tested the inclusion of FS techniques for increasing model performance in ML algorithms. Using the Inter2 approach, we selected 539 ($\sim$7.27%), 69 ($\sim$20.78%) and 82 ($\sim$27.70%) markers for EG1, EG2-S1 and EG2-S2, respectively. For Inter3, 113 ($\sim$1.52%), 8 ($\sim$2.41%) and 15 ($\sim$5.07%) markers were identified. This SNP subsetting approach was beneficial for EG1 (Supplementary Fig. S19A), EG2-S1 (Supplementary Fig. S20) and EG2-S2 (Supplementary Fig. S21); however, there were less pronounced improvements for data from EG2 sites, which was expected because of the limited SSR marker dataset. We considered that, even with increased predictive accuracies, to achieve better results, a wider set of markers would be required. Then, we considered the best strategy for EG2-S1 to be the combination of the Inter2 FS approach with SVM and that for EG2-S2 to be the combination of Inter3 FS with the AdaBoost ML algorithm.

Even though FS approaches boosted prediction accuracies for EG1, when analyzing model performance by calculating the Pearson correlation between the real and predicted BLUPs for each family separately, we observed that this better performance was caused by the predictions coming from the family with the largest number of individuals, which showed a clear inefficiency of the model for the other families. However, when analyzing predictive power within families (Supplementary Fig. S19B), such an approach was not sufficient for obtaining a reliable prediction with this evident data stratification. In this context, different from EG2, we developed an approach specific to datasets similar to EG1, i.e., a methodology with high capabilities to supply accurate predictions, even considering the subpopulation structure present in a dataset.

Considering a genomic prediction problem based on the creation of a regression model for a dataset containing genotypes that belong to different groups of genetically similar individuals, we modeled such a task by dividing the prediction into different stages (Fig. 1) and creating a divide-and-conquer approach for prediction. The basis of such an approach is that closely related genotypes will share QTLs that might not be the same in another group of genotypes. Therefore, we created a different neural network for each biparental population (divide part), coupled with an intrapopulation system of FS and with a different form of hyperparameter estimation. Following this division part, the separated systems were combined using an additional step (the conquer part). To do so, another neural network was created to infer which subpart of the system should be used for prediction.

### Feature selection at the subpopulation level

The selection of subsets of markers was performed according to each EG1 network using the four different tasks: (i) subpopulation prediction, (ii) EG1-Pop1 BLUP prediction, (iii) EG1-Pop2 BLUP prediction, and (iv) EG1-Pop3 BLUP prediction. As expected, each FS strategy returned a different quantity of markers (Table 1). For each subset of markers selected considering Inter2 and Inter3, we evaluated their performance using the ML algorithms selected. Some of the models created for task (i) did not present any mistakes (Supplementary Fig. S22), which was expected due to the subpopulation structure present in the dataset and their evident linear separability. For this task, we considered the most suitable FS strategy to be the Inter2 approach.

**Table 1:** Feature selection strategies performed on the marker dataset considering the intersection among the three methods established (Inter3) and the intersection among at least two out of the three methods established (Inter2).

Prediction scenario	Inter2	Inter3
Subpopulation prediction	224	17
GT1 x PB235	345	20
GT1 x RRIM701	454	62
PR255 x PB217	591	119

For EG1-Pop1 (Supplementary Fig. S23), EG1-Pop2 (Supplementary Fig. S24) and EG1-Pop3 (Supplementary Fig. S25), the best accuracies were observed for the combination Inter2-SVM. However, considering the overall performance with the other algorithms, the best approach for SNP subsetting was Inter3. For this reason, we selected this strategy for the BLUP prediction task. Interestingly, there was no intersection between these three Inter3 datasets in the populations; the only case of overlap was a single SNP marker in Pop2 and Pop3.

From the genomic regions flanking these markers selected for BLUP prediction, we could retrieve several instances of correspondence between rubber tree sequences and CDSs from the *Malpighiales* clade in the Phytozome database. From the 20 markers used in Pop1 for prediction, 62 in Pop2, and 119 in Pop3, we found CDS correspondence for the genomic regions related to 8 (40%), 27 ($\sim$43.55%) and 48 ($\sim$40.32%) SNPs, respectively. Even though there was no obvious complementarity among these markers due to the absence of intersections, we found GO terms with similar biological processes (Supplementary Tables S1–S3), indicating common molecular processes related to these genomic regions.

### Neural network creation

With the marker dataset established through FS for EG1 subtasks, we estimated the best hyperparameter configuration for creating the networks proposed: (i) subpopulation prediction in EG1 (Supplementary Fig. S26), (ii) BLUP prediction in EG1-Pop1 (Supplementary Fig. S27), (iii) BLUP prediction in EG1-Pop2 (Supplementary Fig. S28), and (iv) BLUP prediction in EG1-Pop3 (Supplementary Fig. S29). With the exception of network (i), which is a classification task, for each hyperparameter combination, we evaluated the MAPE and R Pearson coefficient values using the development set to select the best configuration for prediction. For network (i), several hyperparameter combinations returned prediction capabilities without mistakes (Supplementary Fig. S26), which led us to select the configuration with the minimum value for the loss function (Table 2).

**Table 2:** Hyperparameter definition for each one of the created neural networks in experimental groups 1 (EG1) and 2 (EG2) considering (i) the number of neurons selected for the first hidden layer (N-1HL), (ii) the number of neurons selected for the second hidden layer (N-2HL), (iii) the learning rate (LR), and (iv) the activation function (AF).

Neural network	N-1HL	N-2HL	LR	AF
EG1 (Subpopulation Prediction)	45	25	0.005	Rectified linear activation
EG1 (BLUP Prediction in GT1 x PB235)	10	3	0.005	Rectified linear activation
EG1 (BLUP Prediction in GT1 x RRIM701)	30	7	0.005	Rectified linear activation
EG1 (BLUP Prediction in PR255 x PB217)	42	4	0.005	Rectified linear activation

For networks (ii), (iii) and (iv), we selected the best hyperparameter combination by evaluating the plot profiles. We selected the combinations closest to the right corner of the plots (Supplementary Figs. S27–S29), ideally representing the best MAPE and R Pearson coefficient simultaneously. Interestingly, for the four networks, the best activation function was ReLU, and the learning rate was 0.005, only changing the quantity of neurons in the established HLs. An evaluation of the predictive performance of these networks compared to the traditional genomic prediction approaches with k-fold CV built in the development set revealed significant improvement and effective performance in each population, different from the FS performed using these datasets combined (Supplementary Fig. S19).

The network modeled for EG1-Pop1 showed the largest increases (Supplementary Fig. S30), with a mean improvement of 9 times the initial obtained accuracies. EG1-Pop2 (Supplementary Fig. S31) and EG1-Pop3 (Supplementary Fig. S32) showed increases of 7 and 3 times, respectively. In addition to such significant improvements, the models’ performance was also more stable, with the predictive accuracies having a narrow distribution, as observed in the boxplots’ conformations.

### Divide-and-conquer approach

**Figure 2:**
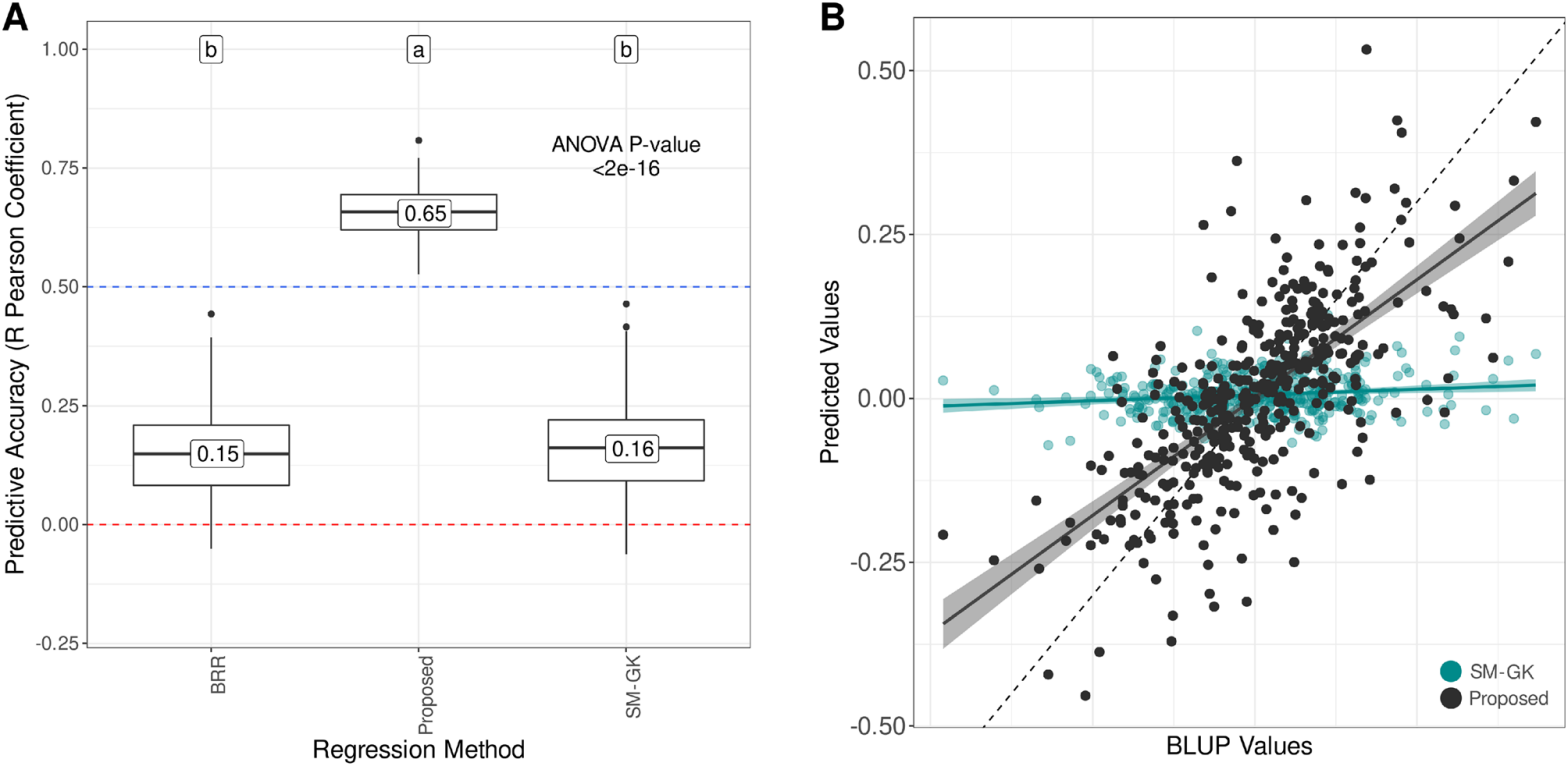
Predictive accuracies for stem circumference BLUP prediction in experimental group 1 (EG1) considering (**A**) a fourfold cross validation (CV) scheme (50 times repeated) and (**B**) a leave-one-out CV strategy. The models used for prediction were a single-environment model with a nonlinear Gaussian kernel (SM-GK), Bayesian ridge regression (BRR), and the proposed strategy using the divide-and-conquer approach. The labels indicate the results from Tukey’s multiple comparison test.

All of the individual networks were combined to create the proposed approach in EG1. Compared with the traditional approaches, this approach showed a mean improvement of 4 times the initial accuracies (Fig. 2A) in the k-fold evaluations. Moreover, BRR and SM-GK presented equivalent performance values. Additionally, when analyzing the performance of the development set for predicting the BLUP values of genotypes from the test set, we found Pearson R coefficients of 0.39, 0.42, and 0.81 for BRR, SM-GK, and the proposed approach, respectively, showing the methodology’s efficiency even for data not in the development set.

As the final step in model evaluation, we performed a LOO CV split to check whether an increase in the training data improves prediction accuracy. By contrasting the real BLUP values with the predicted values, we found R Pearson coefficients of 0.14, 0.16 and 0.68 for BRR, SM-GK, and the proposed approach, respectively. The regression curve clearly indicates the proposed approach’s appropriateness for rubber tree data (Fig. 2B).

## Discussion

GS has emerged as a potential tool for application in plant breeding programs^34–38,94,95^. In rubber tree, previously obtained results^2,25^ have demonstrated the potential of such a technique for reducing breeding cycles. Because of the strong commercial rubber demand, there have been many economic incentives for rubber tree production in more environments beyond its natural range^1,3^. Considering the difficulty of achieving ideal conditions for cultivating *H. brasiliensis* and the rubber demand, the development of more efficient varieties is needed. However, *Hevea*’s long life cycle considerably reduces breeding efficiency^15^. Therefore, the application of GS in rubber tree represents an alternative for achieving the desired rubber production in less time by replacing clone trials and reducing the long period of phenotypic evaluation^2^.

The main objective of rubber tree breeding programs is to increase latex production with rapid growth^4^. Increased SC development can be associated with several rubber tree characteristics, such as growth^96^, latex production^25^, and drought resistance^97^. Due to the high versatility of SC in evaluating rubber trees^98–101^, we proposed to develop more effective models for predicting this trait, providing a method to be incorporated into the estimation of tree performance. The lack of high genotype variability in the datasets used represents a real scenario for rubber tree breeding programs^25^, which face the difficulty of generating a population^2^. In addition to the within-family approach suggested for GS with full-sib families by^2^, the use of interconnected families is a common strategy for perennial species^22,102,103^.

Using these dataset configurations, we evaluated ML algorithms as a more accurate methodology for predicting SC, a complex trait^2^ obtained a mean accuracy for rubber production in a CV0 scenario of 0.53, which increased to 0.56 when selecting a set of markers based on heterozygosity values. In a CV1 scheme, the mean values ranged between 0.33 and 0.60. In the proposed work, we observed even lower accuracies when using SC instead of rubber production, which is in accordance with the findings of^25^. In^25^, the authors achieved mean accuracies ranging between 0.19 and 0.28 in a CV1 scenario, contrasted with a CV2 scheme with values ranging between 0.84 and 0.86. For unknown tested genotypes, the predictive accuracies in rubber tree are low, and the inclusion of GS in *Hevea* breeding programs is therefore still not feasible.

Using the traditional approaches for prediction, we achieved LOO configurations of 0.14 and 0.16 for the BRR and SM-GK approaches, respectively, which is similar to what^25^ observed. The BRR and SM-GK methodologies were selected to represent a parametric and a semiparametric approach^104^. Different from BRR, which estimates marker effects, SM-GK estimates genotype effects through a relationship matrix obtained with a reproducing kernel^76^. Even though^25^ found similar results when using a linear and a nonlinear kernel for the estimation of the genomic relationship matrix^105^, considered GK to have a more flexible structure and a higher associated performance. Therefore, considering these findings together with the fact that no significant differences have been found among statistical models for GS^31,46,47^, we selected only these two statistical models for predictive evaluation.

Even though some previous attempts did not reveal significant differences in employing ML in GS compared with traditional linear regression methodologies^32,33,39,52,106^, this is not what we observed in our study, which corroborates the findings of^23,31,107,108^. This discrepancy may be explained by the different strategies used in the ML algorithms, especially distinct neural network architectures, training methodologies, and CV scenarios. The design of neural network architectures is an important step in using deep learning for prediction because differences in the definition of topologies can lead to decreased accuracies^31^.

Several factors are known to influence prediction accuracy in GS, such as the relationship between the individuals used to train models and those that will be predicted^21^, the size and structure of the populations used^24^, the trait heritability^109^, the marker density^110^, and the linkage disequilibrium (LD) between the set of markers used and the associated QTLs^111^. This last aspect is especially critical in the datasets employed because of the limited set of markers obtained through GBS and SSR genotyping. Considering the reduced accuracies obtained with the CV1 technique already described in^2,25^, it was expected that when using a K-fold strategy, the same observations would be found for the traditional regression models.

One of the main challenges in GS is the high dimensionality of the features in the datasets because the number of SNPs is much larger than the number of phenotypic observations^112^ (‘large *p*, small *n*’ problem). Although a greater saturation of markers enables an increase in the probability of finding LD, a larger number of markers in the same LD block does not contribute to better prediction performance^110^. In this context, FS techniques may be an alternative strategy for building a predictive model, considering that not all markers are related to a specific phenotype^113^ and that the quantity required for this task directly depends on the complexity and genetic architecture of the traits used^110^. Therefore, like^23,82,114–118^, and^119^, we decided to test the prediction improvements by using an FS technique to enhance network performances.

Subset selection showed improvements for EG2 (Supplementary Figs. S20, S21); however, there were no sizable improvements because of the genetic complexity of SC^120^ and the low density of SSR markers^121^. In EG1, although an overall improvement in prediction accuracy was observed (Supplementary Fig. S19), when evaluating the intrapopulation predictive accuracy, we observed clear inefficiency of the approach, probably caused by the different allele substitution effects between the three subpopulations employed^111^. In such a scenario with unbalanced interconnected families, novel approaches are needed, and in this work, we have proposed the use of a divide-and-conquer strategy.

In computer science, the divide-and-conquer paradigm is based on the principle that if a problem is not simple enough to be solved directly, it can be divided into subproblems, and their results can be combined^122^. In our prediction task, the BLUPs of the populations could not be properly predicted together; thus, we separated the problem into different networks for prediction, combining the strategy into a single network structure. Such an approach has already been applied to the development of neural network architectures^123–126^; however, such a formulation has not been explored in genomic prediction. In addition to increasing prediction accuracies, such an approach can reduce the time required for network training and hyperparameter estimation^124^, supply superior model interpretability without loss of performance^127^, and be used in combination with other models^128^, including traditional genomic prediction methods. Considering that in genomic prediction, most of the scenarios include different population structures, such a paradigm can benefit the application and development of GS strategies.

In our dataset, most of the observed variance within SNP markers was caused by population structure, which is clearly shown by the PCA results (Supplementary Fig. S14). As this strong variability can be associated with several genomic regions and influence various traits differently and simultaneously in the populations^129^, we hypothesize that traditional genomic prediction models are not capable of capturing these interpopulation differences related to SC QTLs. This is the main reason why performing FS on these unbalanced datasets together was not a promising strategy in our study. As intrapopulation QTLs are not transferable to other populations, the main effects on phenotypic variation are specific to the within-population genetic structure^130^. In this sense, the prediction task in single populations can be seen as simpler than that in multiple populations^131^, which was the basis for developing the divide-and-conquer strategy. Considering the specific effects of causal genetic variants within populations^132,133^, we tried to incorporate such factors into separate networks with their specific hyperparameter optimization processes.

Interestingly, FS steps performed in the three different populations of EG1 returned different markers, but these markers were putatively associated with genes acting in similar biological processes. GO mRNA splicing was found in the intersection set of markers selected for the three populations. The occurrence of genetic variation related to such a regulatory process may influence the transcription of diverse mRNAs from the same gene in different ways. Such diversity of molecules may be related to differences in phenotypic performance, leading to increased plant capabilities^134–136^. Additionally, base-excision repair was found in both Pop1 and Pop3, which represents a very important defense pathway for maintaining genomic integrity^137^ and is clearly essential for rubber tree growth and development^138^. Due to the increased quantity of individuals in Pop2 and Pop3, more GO categories were found, including important processes for plant growth, such as response to different types of stress and several metabolic processes^120^.

Different studies have reported the use of deep learning for genomic prediction with various datasets, including for humans^23,113^, sows^107^, and plant species such as soybean^108^, wheat^31–33,39,52^, maize^33^, and strawberry and blueberry^106^. Even though all of these studies used deep learning, the neural network creation approaches were not the same; some of them included architectures of convolutional neural networks (CNNs)^106,107,113^, while others included MLPs^32,33,39,52^ or both approaches^23,31,108^. There is no consensus on the efficiency of neural networks for genomic prediction; however, we decided to use such an architecture for combining multiple training processes into a single predictive structure.

For each of the neural network architectures, we employed an MLP structure. We did not include convolutional operations because of the reduced quantity of markers obtained through FS. Additionally, CNNs were developed for extracting unknown patterns from the dataset, and as we hypothesized that FS operations might work as indicators of QTL regions, such operations would not be necessary. To define the most promising network architecture, we used a grid search, testing different combinations of hyperparameters as already performed in relation to GS strategies^32,33,39,52^. Although other researchers have used the ‘trial and error’ approach to define the network topology^139^, we preferred to develop a strategy that could be replicated in other predictive scenarios, especially with other traits and crops.

The approximation of functions through neural networks was supported first based on^140^ and later on^141^, which extended the theorem of^140^, proving that any continuous function can be represented by a neural network with one HL containing $2n+1$ nodes (*n* features) and a more complex activation function than that usually employed by current researchers^92^. It has already been proven that one HL is capable of universal approximation by using a complex activation function^91,142–145^; however, when using regular functions, such as sigmoid and ReLU functions, there is reduced efficiency of such networks. In this context^146^, suggested that two HLs could be a solution for this reduced efficiency. In addition, the usage of an additional HL can substantially reduce the total number of required nodes for a satisfactory predictive capability^92^, and it has already been shown that some problems can be solved only by the use of two HLs^143,147,148^. In practical situations, a neural network architecture with two HLs generalizes better than that with one and has been considered a superior approach^143,149^. Therefore, in our study, we decided to include two HLs in our proposed architecture, representing a network with more complex training complexity^150^.

Concerning the quantity of hidden neurons in a neural network, many researchers have developed different strategies, aiming at increasing accuracy and prediction while decreasing errors^139^.^91^ has already proven that in a network architecture with two HLs, the number of nodes required to achieve a reasonable predictive accuracy with *m* samples and *q* output neurons is $\sqrt{(q+2)m}+2\sqrt{m/(q+2)}$ in the first HL and $q\sqrt{m/(q+2)}$ in the second HL. However, the quantity of suggested nodes tends to lead to overfitting of the training data with any arbitrary small error^139^, and considering the capability of predicting unknown data, these values can be considered the maximum number of nodes in an artificial neural network structure^92^. The lower bound for hidden neurons was already proposed by^151^, which can be useful for accelerating the learning speed, but there was no evidence on separating this quantity across HLs, and the study was based on an MLP with 3 HLs^139^. Thus, in our architecture definition, we decided to test a large quantity of neurons, considering the findings of^91^, as our upper bound.

The created network coupling the population-specific architectures could increase the initial prediction capabilities by more than four times. Such an improvement represents the first attempt to develop a ML strategy for genomic prediction in rubber tree, with a high potential to be adapted to other species with the same data configuration. Considering a broader scenario with distantly related genotypes belonging to a population with undefined structure, this same approach could be applied. Instead of relying on the predefined stratification, clustering analyses could be performed and used for the divide part. Such a practice is already common in breeding, i.e., taking advantage of population structure for model prediction through multivariate techniques^152–155^. Taking into account the importance of such group configuration in the differentiation of multiple traits^156–158^, the strategy developed represents a promising approach for several plant species with a difficult prediction scenario.

The use of GS in rubber tree can optimize breeding programs, and the incorporation of ML techniques can be seen as a new possibility for building more robust models with higher associated prediction capabilities. By using data from rubber tree breeding programs, we were able to generate promising predictive results for a highly complex trait and a novel strategy for prediction, which has significant potential to enhance selection efficiency, and reduce the length of the selection cycle. Although our results confirmed the efficiency of the methodology proposed for rubber tree data, to properly evaluate the full potential of the method in other species and broader scenarios, our approach should be investigated in further studies with more genetically diverse populations in contrasting environments.

## Supplementary Information

Supplementary Information 1.

Supplementary Information 2.

## Data Availability

All the genotypic data from this study are available in the Supplementary Material and under NCBI accessions PRJNA540286 (ID: 5440286) (GT1 × PB235 and GT1 × RRIM701) and PRJNA541308 (ID: 541308) (PR255 × PB217).
