# Supplementary material for: A divide-and-conquer approach for genomic prediction in rubber tree using machine learning
Source: Sci Rep. 2022 Oct 26;12:18023. doi: 10.1038/s41598-022-20416-z (PMC9605989; doi:10.1038/s41598-022-20416-z)
Supplement: Supplementary file 1 — Supplementary Information 1. [file 41598_2022_20416_MOESM1_ESM.pdf]

SUPPLEMENTARY RESULTS

A divide-and-conquer approach for genomic prediction in rubber tree using machine learning

SUPPLEMENTARY TABLES

Supplementary Table S1. Gene Ontology (GO) terms summarized through the Revigo tool for the 20 SNPs selected by the feature selection techniques employed on the individuals of the GT1 x PB235 population.

GO Term	Name	Frequency (%)
GO:0006284	base-excision repair	0.298165436998951
GO:0000398	mRNA splicing, via spliceosome	0.436433865037325

Supplementary Table S2. Gene Ontology (GO) terms summarized through the Revigo tool for the 62 SNPs selected by the feature selection techniques employed on the individuals of the GT1 x RRIM701 population.

**GO Term**	**Name**	**Frequency (%)**
GO:0006355	regulation of transcription, DNA-templated	10.0330757015203
GO:0006468	protein phosphorylation	4.23350556337001
GO:0006950	response to stress	4.70454267943585
GO:0007018	microtubule-based movement	0.349985633268822
GO:0006886	intracellular protein transport	1.23288694299588
GO:0000103	sulfate assimilation	0.084209367967923
GO:0000398	mRNA splicing, via spliceosome	0.436433865037325
GO:0016192	vesicle-mediated transport	1.40900125690309
GO:0006810	transport	18.3767799586826
GO:0000160	phosphorelay signal transduction system	2.76884251732889
GO:0006396	RNA processing	3.83107599133337
GO:0006412	translation	5.34694375263645
GO:0006351	transcription, DNA-templated	2.0006354738138

Supplementary Table S3. Gene Ontology (GO) terms summarized through the Revigo tool for the 119 SNPs selected by the feature selection techniques employed on the individuals of the PR255 x PB217 population.

GO Term	Name	Frequency (%)
GO:0006468	protein phosphorylation	4.23350556337001
GO:0008152	metabolic process	62.7747296551307
GO:0009611	response to wounding	0.102041794217188
GO:0010223	secondary shoot formation	0.002263648270697
GO:0006886	intracellular protein transport	1.23288694299588
GO:0009694	jasmonic acid metabolic process	0.00696443610291
GO:0022900	electron transport chain	1.71627498667009
GO:0071704	organic substance metabolic process	55.4341066032933
GO:0005975	carbohydrate metabolic process	5.96269325897111
GO:0000398	mRNA splicing, via spliceosome	0.436433865037325
GO:0006788	heme oxidation	0.016089251851028
GO:0009116	nucleoside metabolic process	0.779120471856656
GO:0006260	DNA replication	1.52888373885959
GO:0006465	signal peptide processing	0.134129421528815
GO:0000160	phosphorelay signal transduction system	2.76884251732889
GO:0007165	signal transduction	7.31364928685992
GO:0034968	histone lysine methylation	0.101822864731154
GO:0016571	histone methylation	0.115326270199963
GO:0006355	regulation of transcription, DNA-templated	10.0330757015203
GO:0009408	response to heat	0.187738230392138

GO:0043039	tRNA aminoacylation	1.06491019677713
GO:0006419	alanyl-tRNA aminoacylation	0.058288942970439
GO:0006633	fatty acid biosynthetic process	0.793193920138159
GO:0006979	response to oxidative stress	0.582480485947453
GO:0006486	protein glycosylation	0.522109647487177
GO:0006487	protein N-linked glycosylation	0.113839201992936
GO:0006284	base-excision repair	0.298165436998951
GO:0009873	ethylene-activated signaling pathway	0.037081698040226
GO:0006505	GPI anchor metabolic process	0.153101933403468
GO:0009864	induced systemic resistance, jasmonic acid mediated signaling pathway	0.001012032529782

SUPPLEMENTARY FIGURES

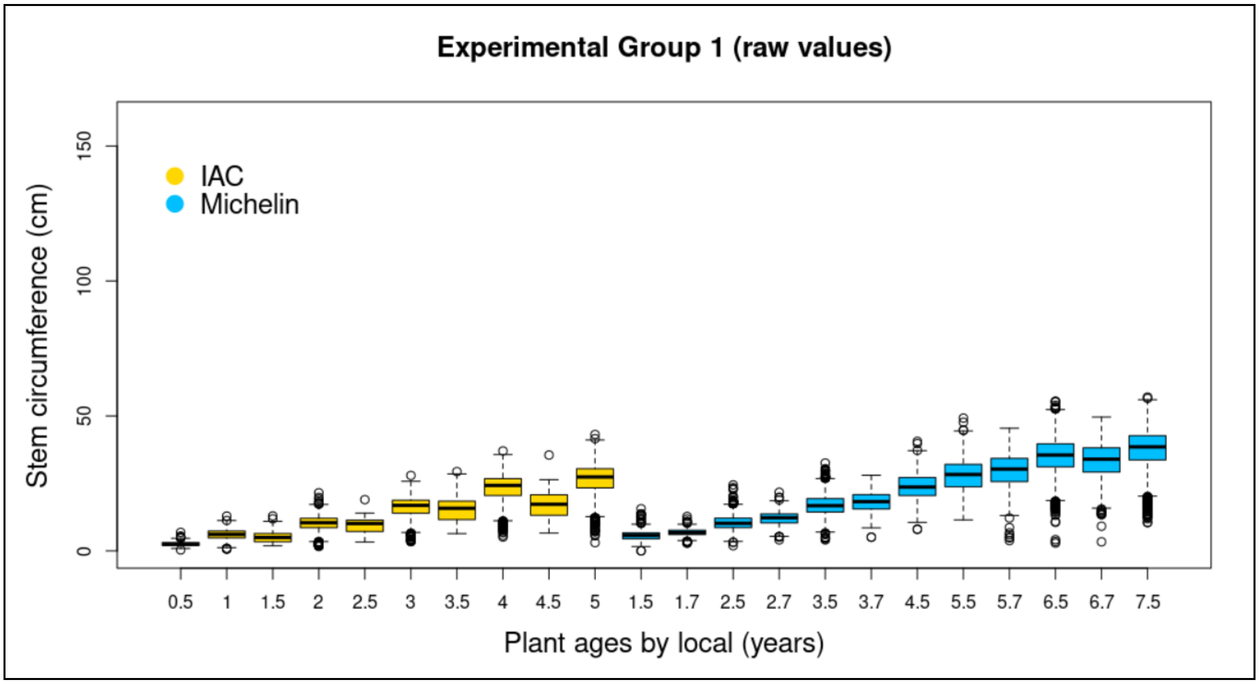

Supplementary Fig. S1. Distribution of raw phenotypic data in Experimental Group 1 (EG1) for stem circumference (SC) separated according to the location and the year of measurement.

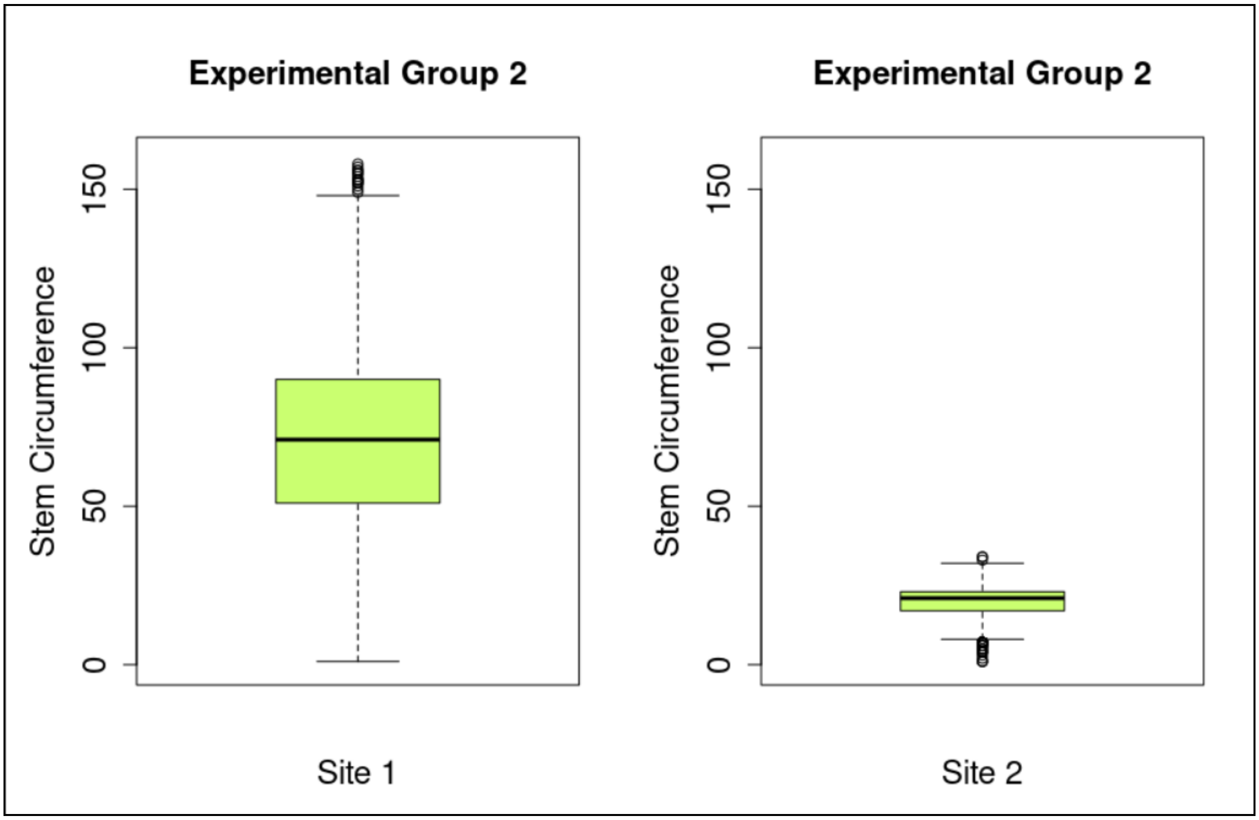

Supplementary Fig. S2. Distribution of raw phenotypic data in Experimental Group 2 (EG2) for stem circumference (SC) at sites 1 and 2.

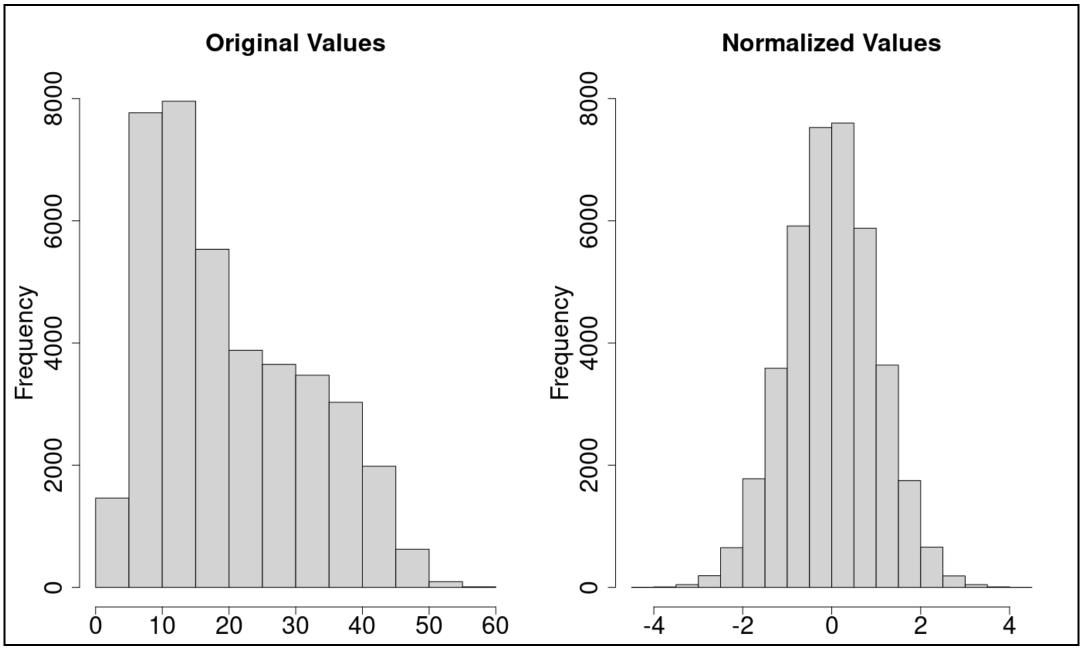

Supplementary Fig. S3. Distribution of trait data before and after normalization in Experimental Group 1 (EG1) for stem circumference (SC).

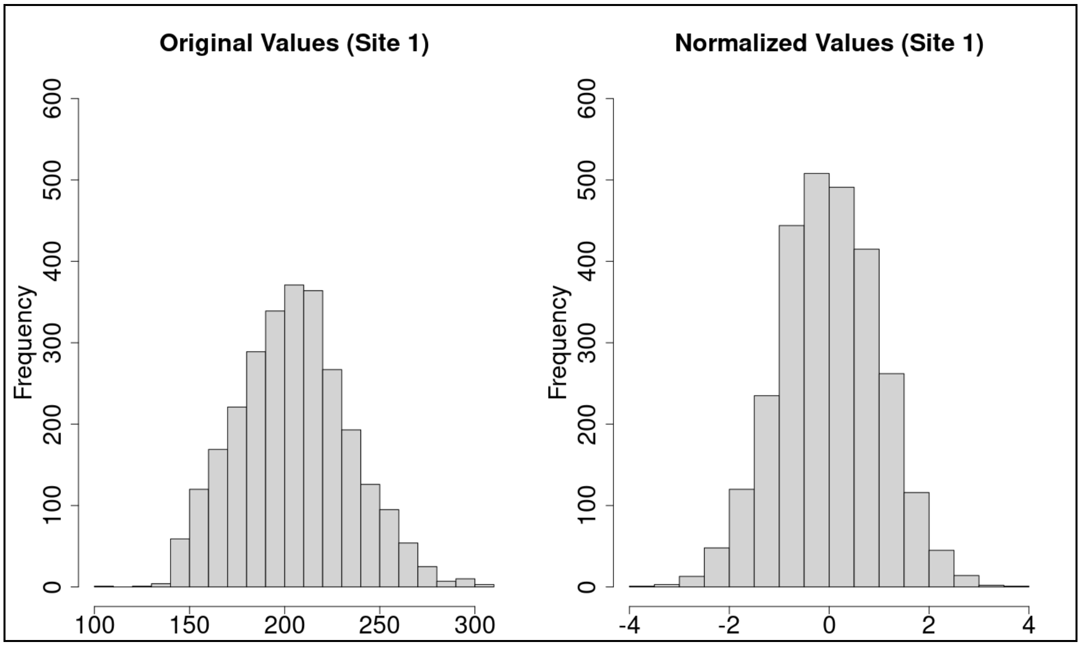

Supplementary Fig. S4. Distribution of trait data before and after normalization in Experimental Group 2 (EG2) for stem circumference (SC) at site 1 (S1).

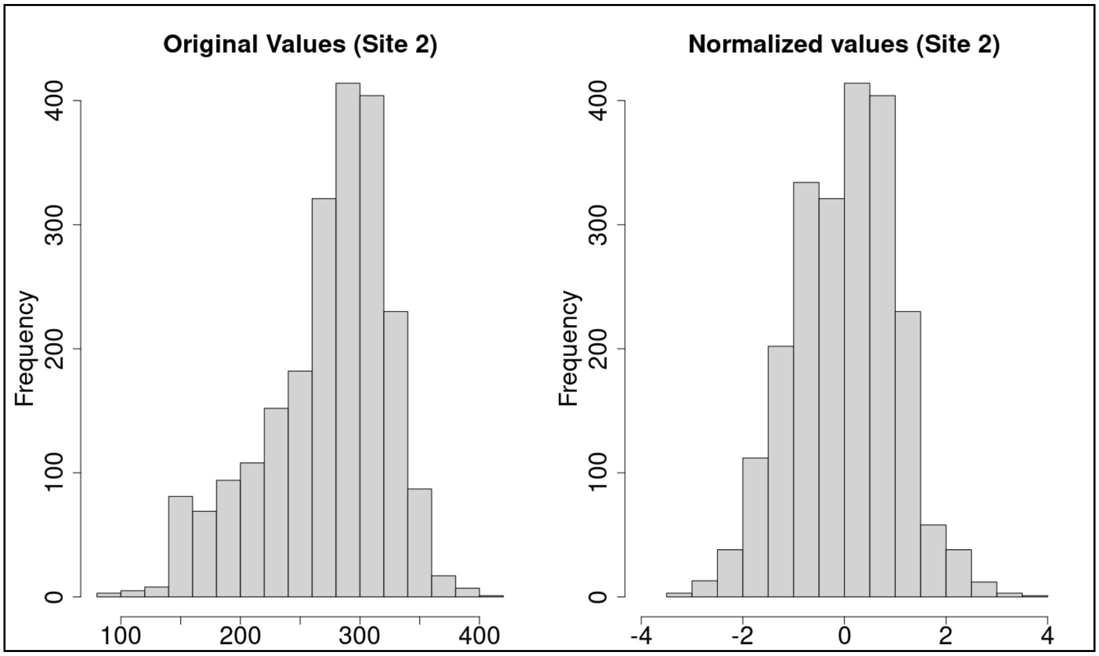

Supplementary Fig. S5. Distribution of trait data before and after normalization in Experimental Group 2 (EG2) for stem circumference (SC) at site 2 (S2).

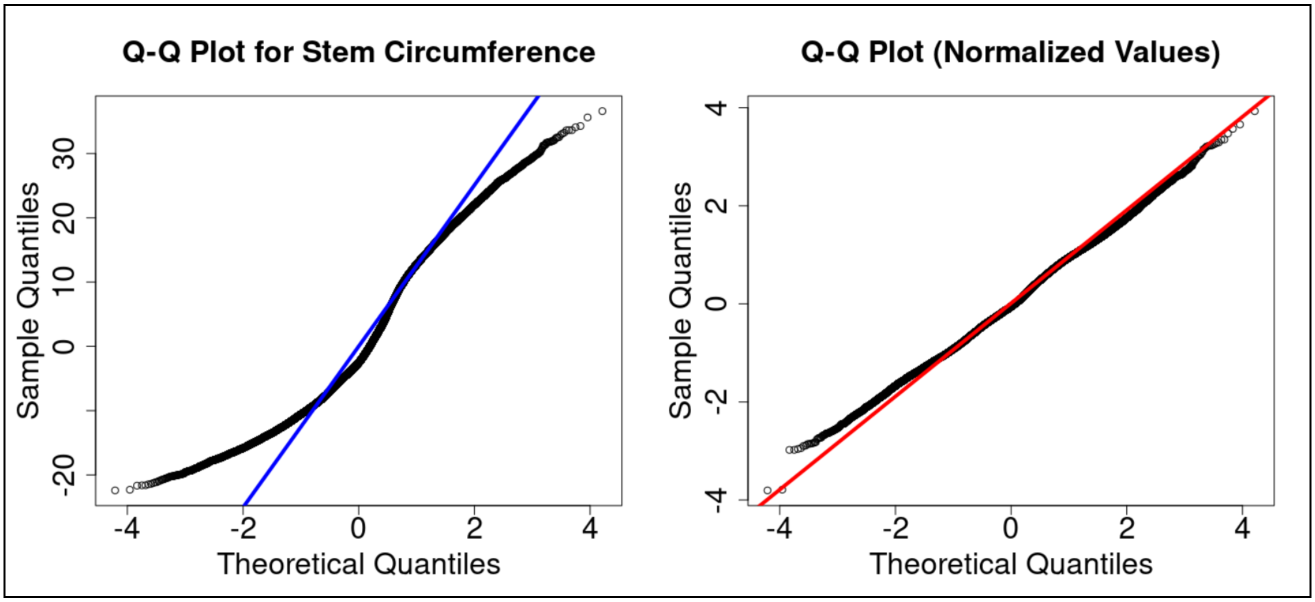

Supplementary Fig. S6. Quantile-quantile (Q-Q) plots for the residual distributions of the mixed models created for stem circumference (SC) in Experimental Group 1 (EG1).

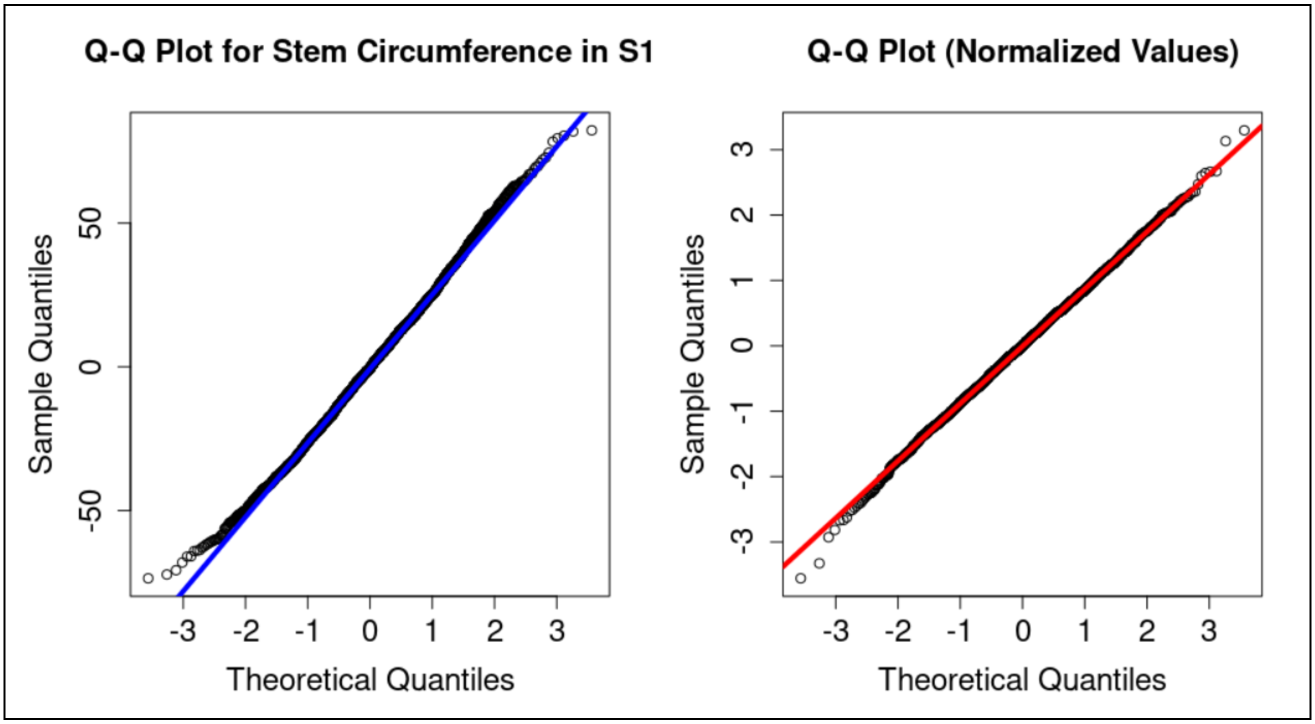

Supplementary Fig. S7. Quantile-quantile (Q-Q) plots for the residual distributions of the mixed models created for stem circumference (SC) in Experimental Group 2 (EG2) at site 1 (S1).

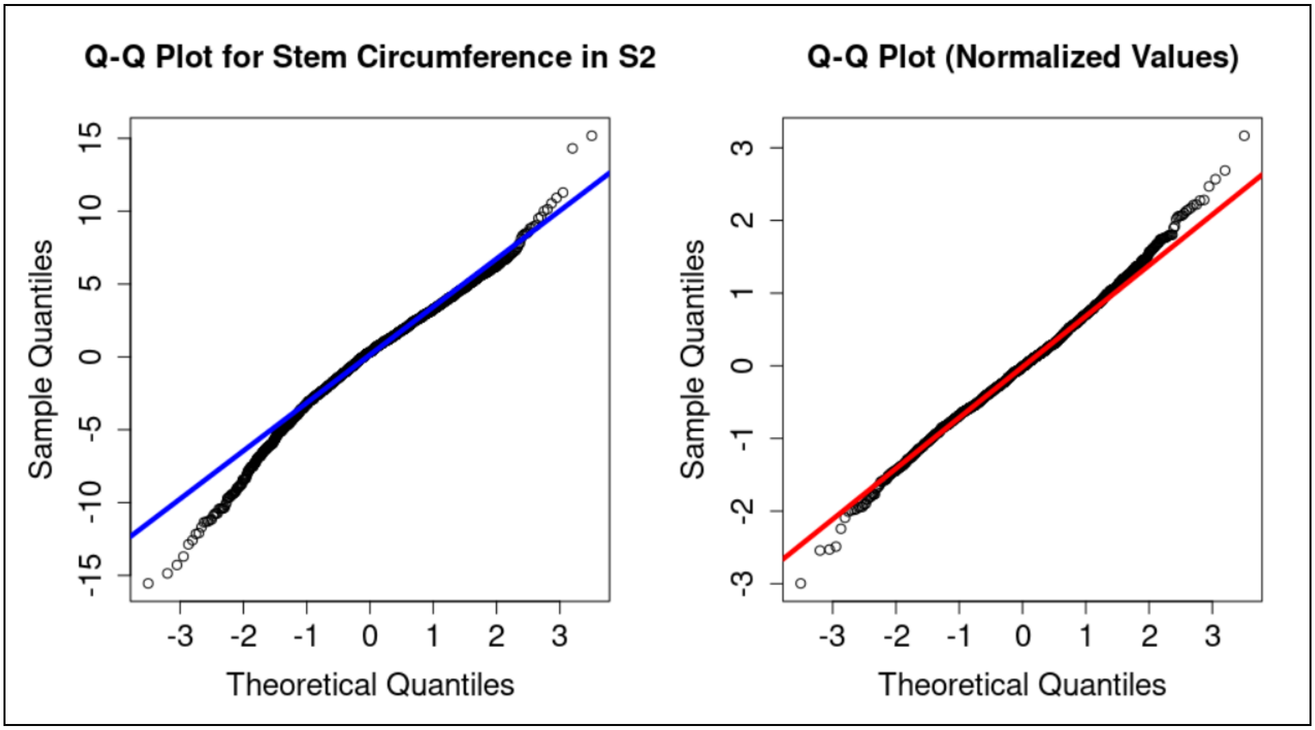

Supplementary Fig. S8. Quantile-quantile (Q-Q) plots for the residual distributions of the mixed models created for stem circumference (SC) in Experimental Group 2 (EG2) at site 2 (S2).

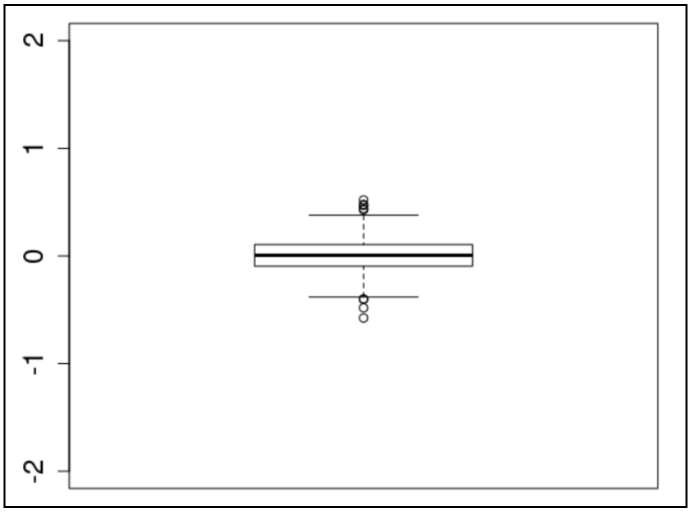

Supplementary Fig. S9. Distribution of estimated BLUP (B) values in Experimental Group 1 (EG1) for stem circumference (SC).

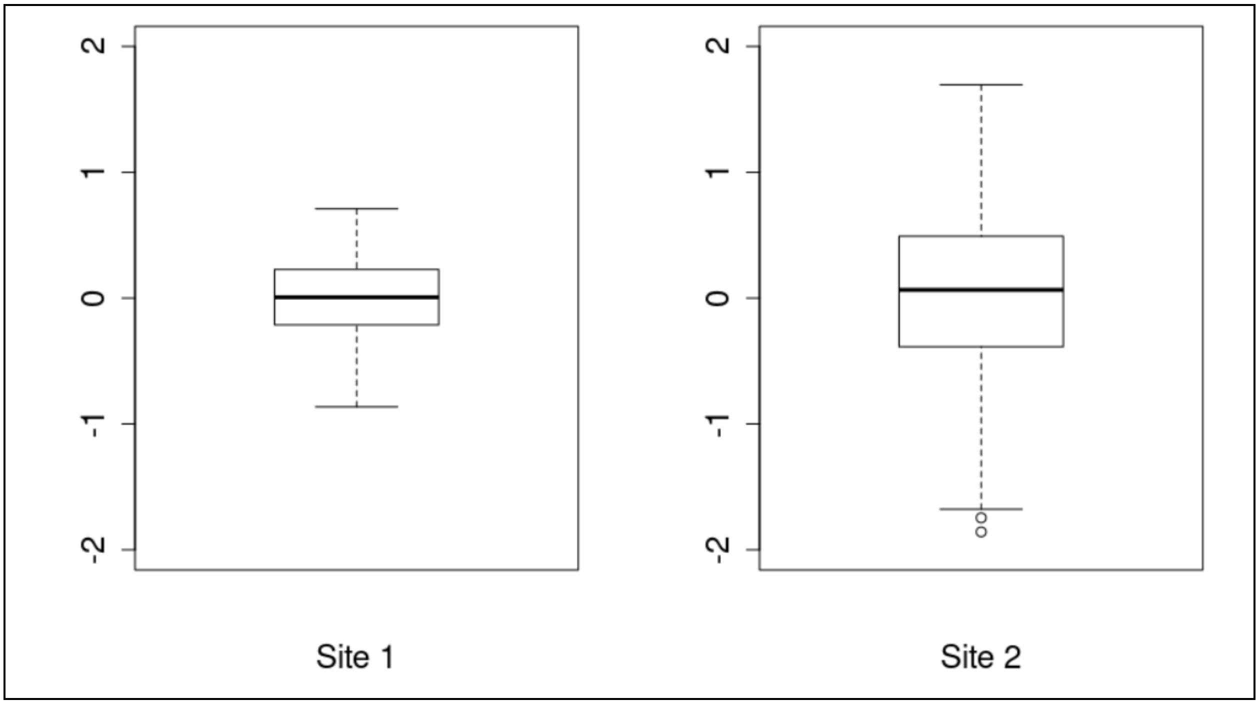

Supplementary Fig. S10. Distribution of estimated BLUP (B) values in Experimental Group 2 (EG2) for stem circumference (SC) at sites 1 and 2.

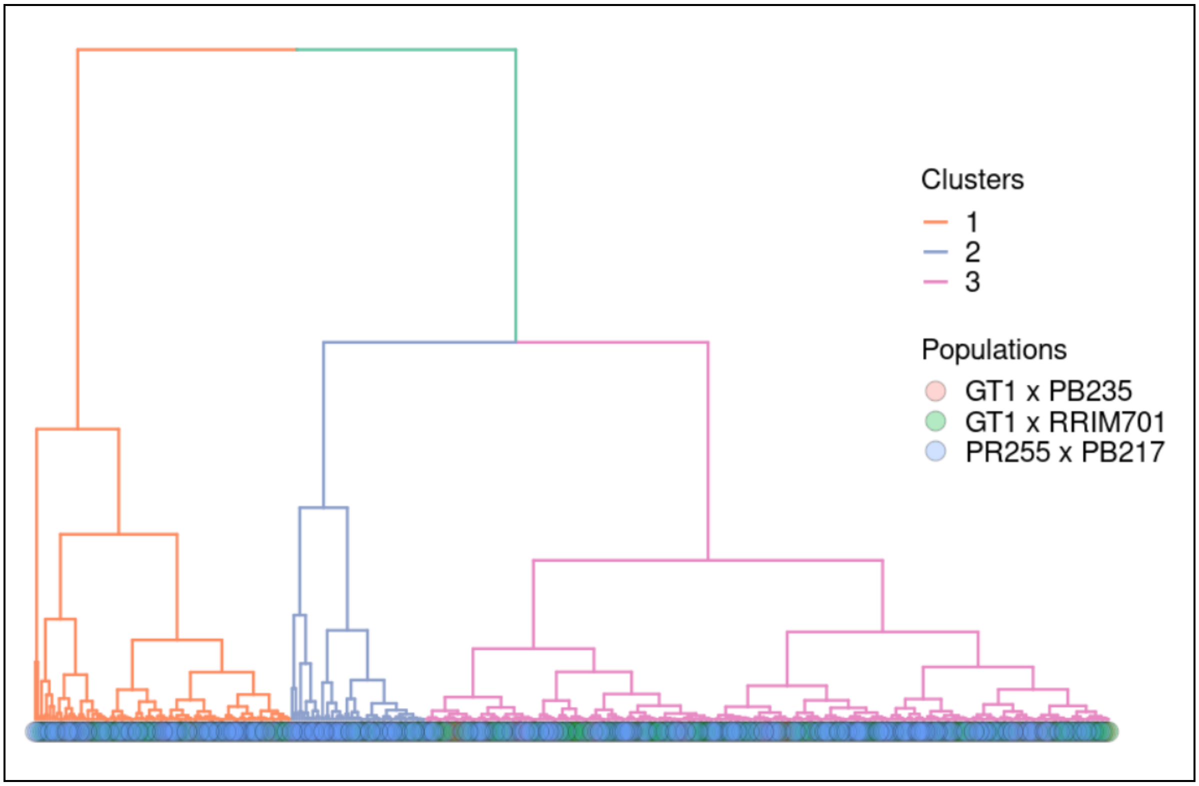

Supplementary Fig. S11. Constructed dendrograms colored based on the identified phenotypic groups for the BLUP (B) values in Experimental Group 1 (EG1) for stem circumference (SC).

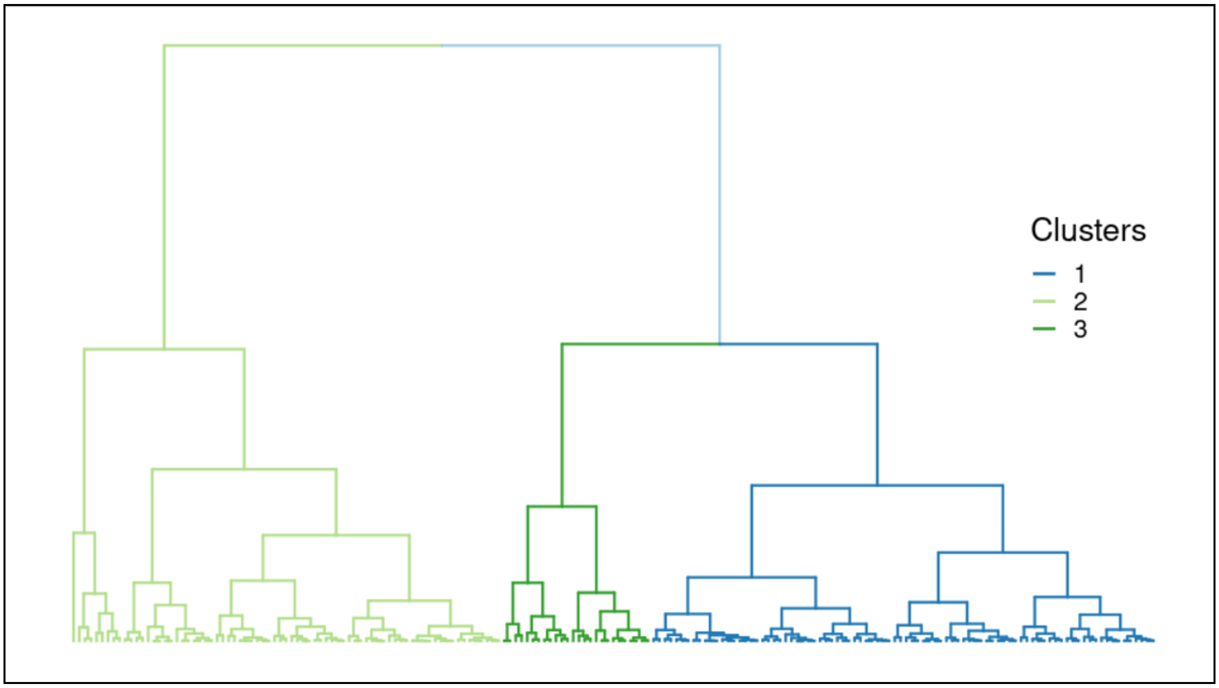

Supplementary Fig. S12. Constructed dendrograms colored based on the identified phenotypic groups for the BLUP (B) values in Experimental Group 2 (EG2) for stem circumference (SC) at site 1 (S1).

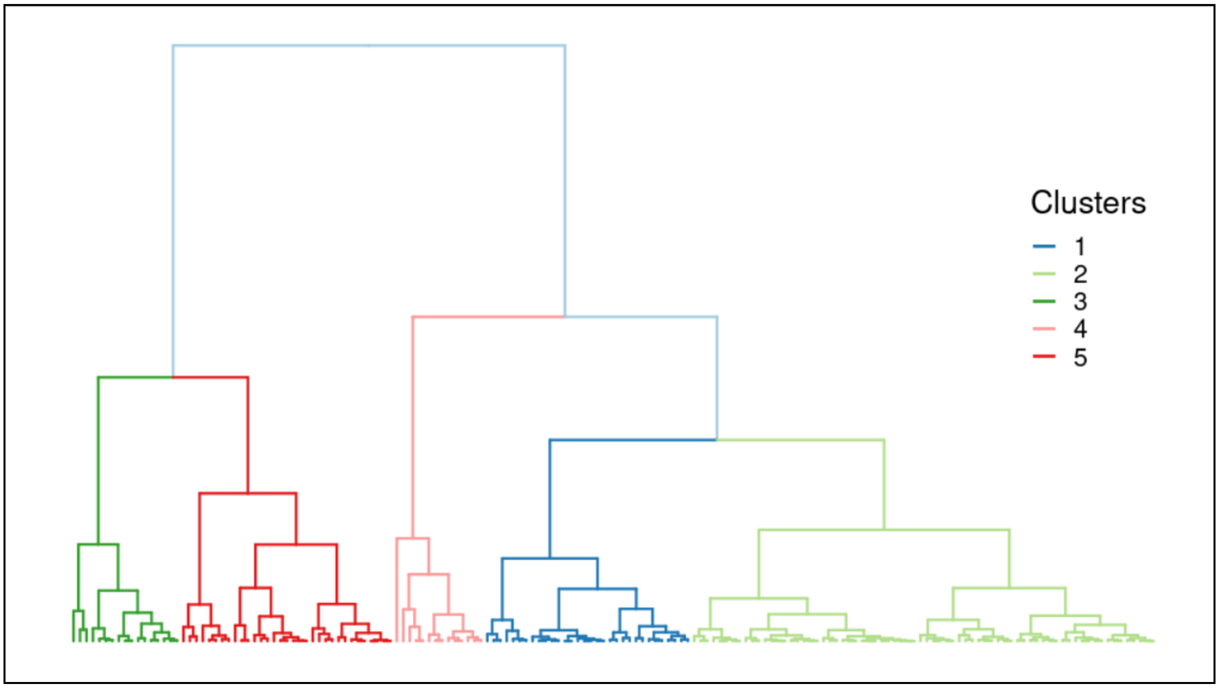

Supplementary Fig. S13. Constructed dendrograms colored based on the identified phenotypic groups for the BLUP (B) values in Experimental Group 2 (EG2) for stem circumference (SC) at site 2 (S2).

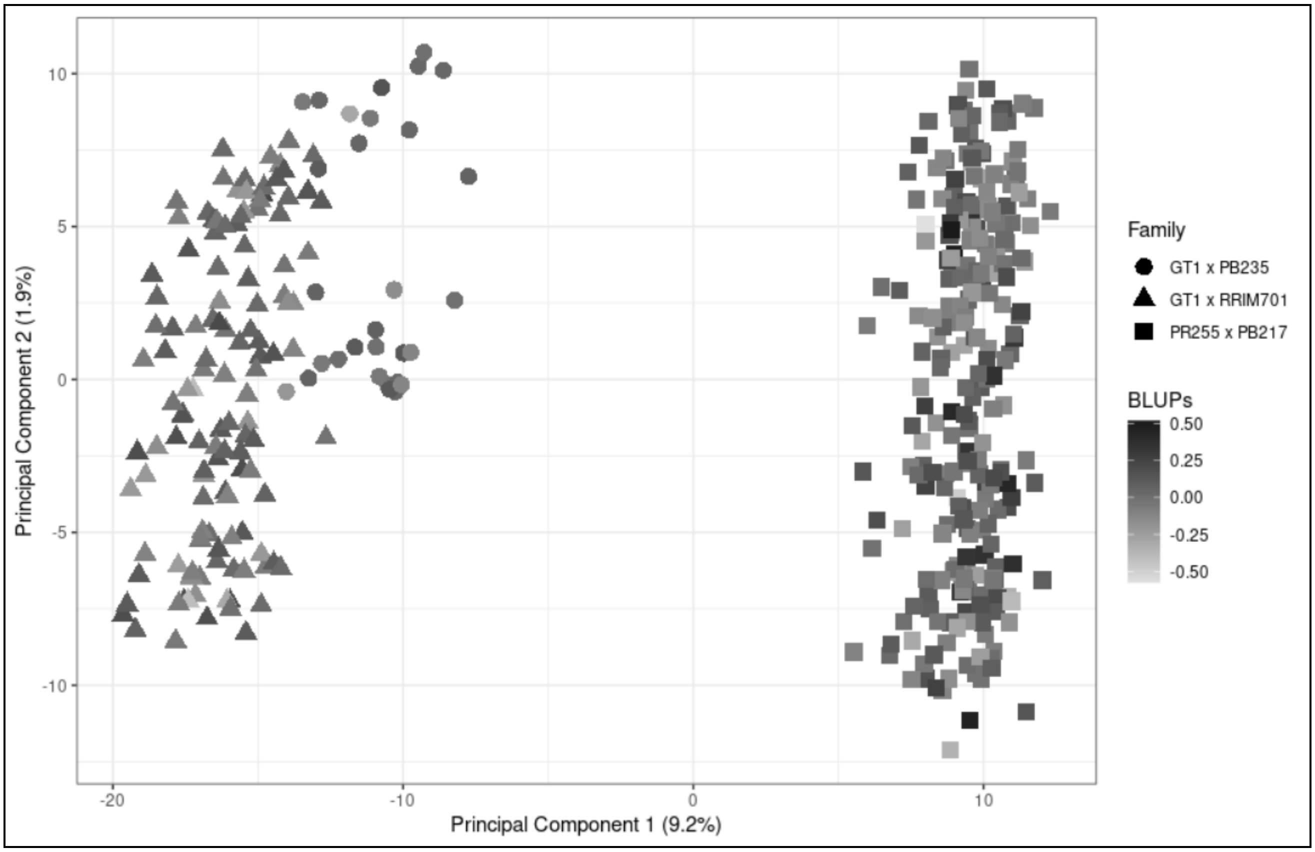

Supplementary Fig. S14. Principal component analysis (PCA) performed on SNP markers and colored based on stem circumference (SC) BLUPs. Each point represents an individual of experimental group 1 (EG1), belonging to a specific family (point shapes) and with a different BLUP value (intensity of gray).

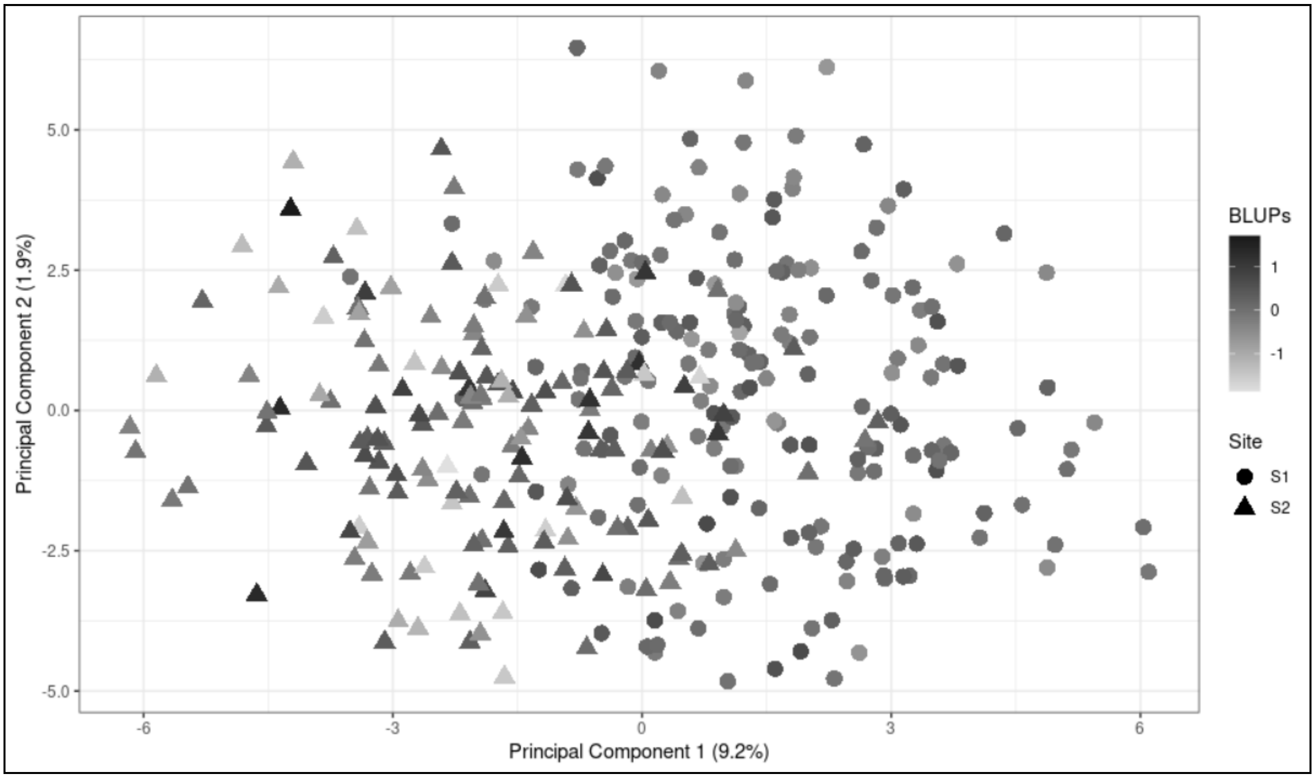

Supplementary Fig. S15. Principal component analysis (PCA) performed on SSR markers and colored based on stem circumference (SC) BLUPs. Each point represents an individual of experimental group 2 (EG2) at sites 1 (S1) and 2 (S2) with a different BLUP value (intensity of gray).

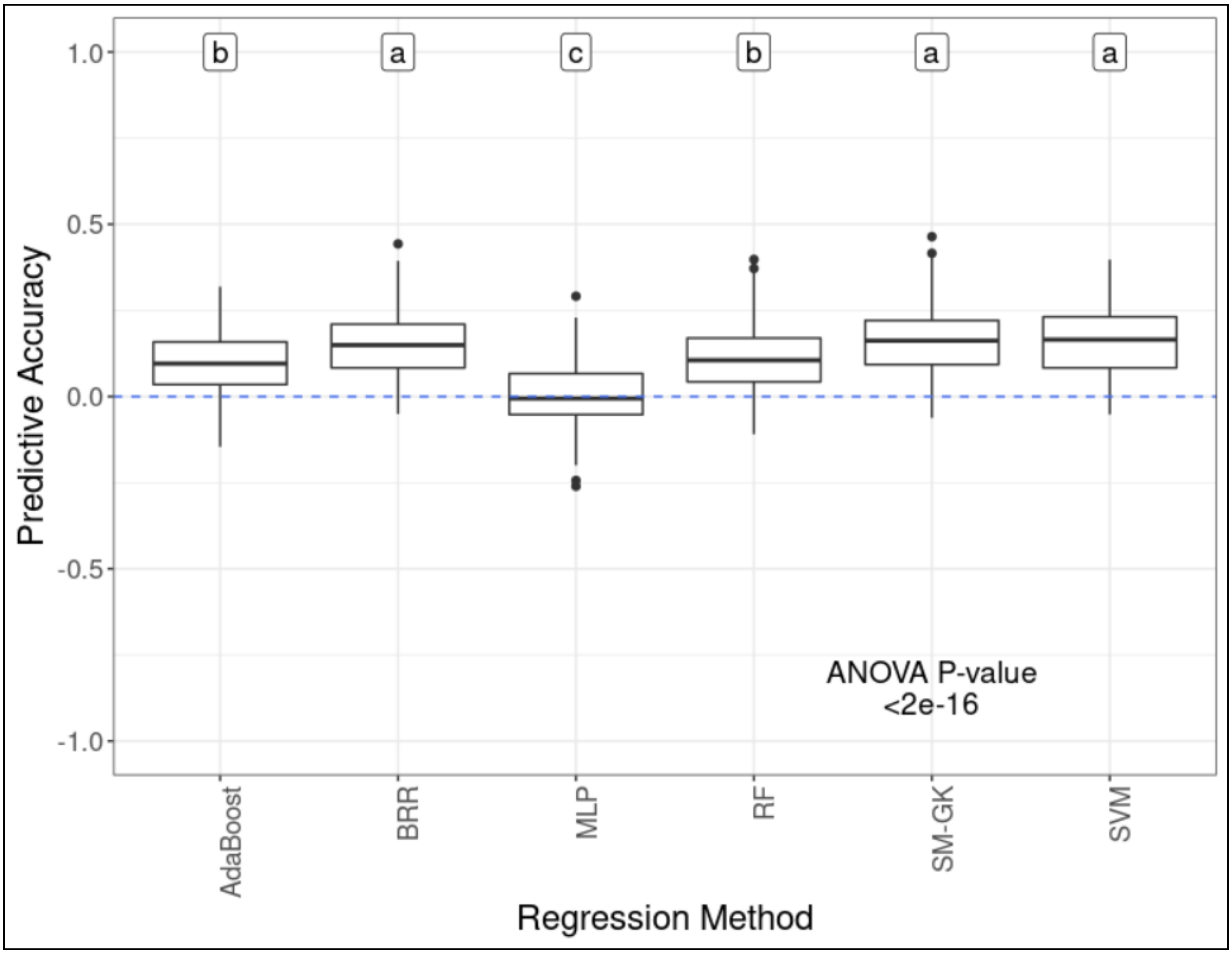

Supplementary Fig. S16. Predictive accuracies for stem circumference BLUP prediction in experimental group 1 (EG1) considering a 4-fold cross validation scheme (repeated 50 times), a single-environment model with a nonlinear Gaussian kernel (SM-GK), Bayesian ridge regression (BRR), and several machine learning algorithms for regression (AdaBoost, multilayer perceptron (MLP), random forest (RF), and support vector machine (SVM)). The letters at the top indicate the results from Tukey's multiple comparison test.

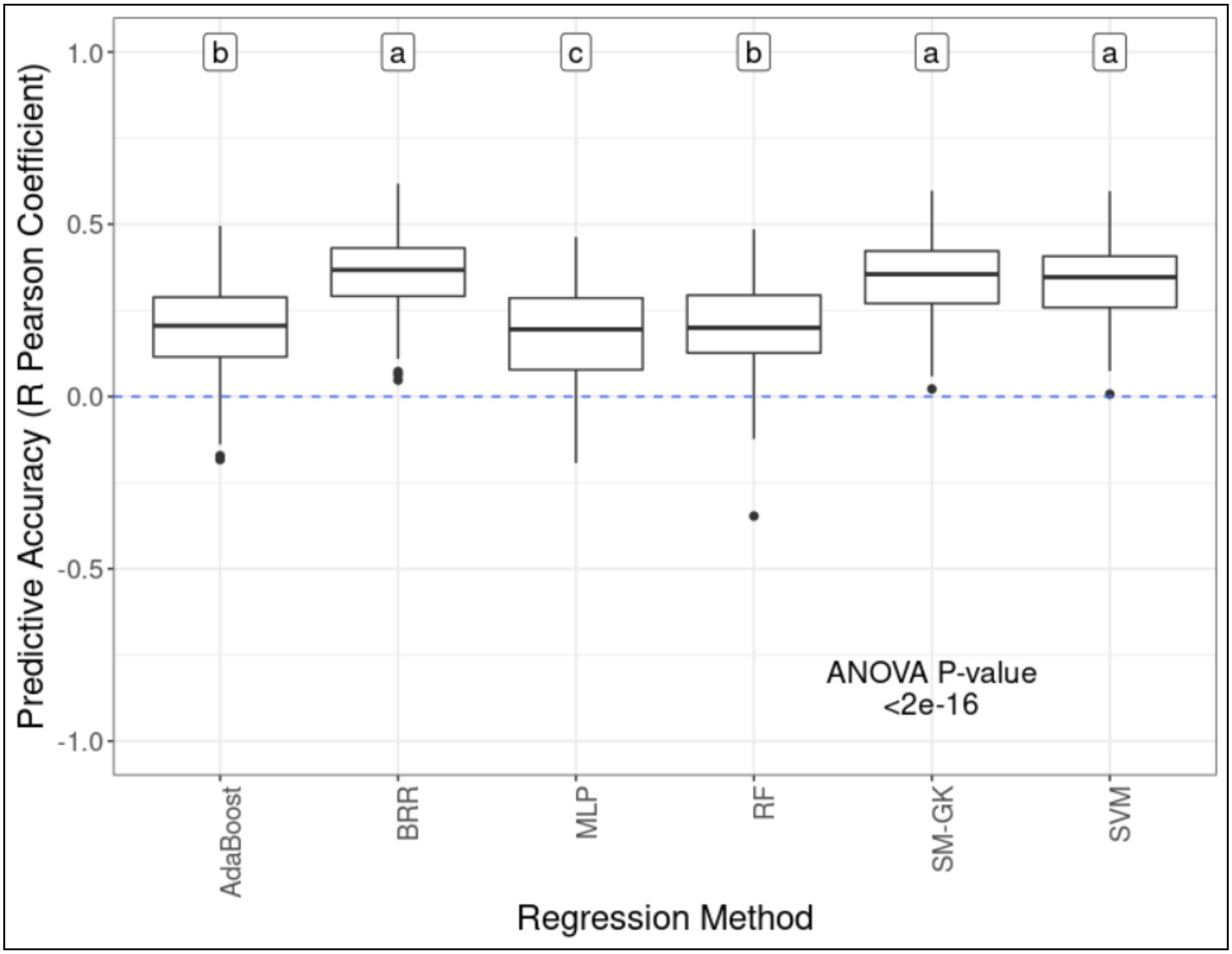

Supplementary Fig. S17. Predictive accuracies for stem circumference BLUP prediction in experimental group 2 (EG2) at site 1 (S1) considering a 4-fold cross validation scheme (repeated 50 times), a single-environment model with a nonlinear Gaussian kernel (SM-GK), Bayesian ridge regression (BRR), and several machine learning algorithms for regression (AdaBoost, multilayer perceptron (MLP), random forest (RF), and support vector machine (SVM)). The letters at the top indicate the results from Tukey's multiple comparison test.

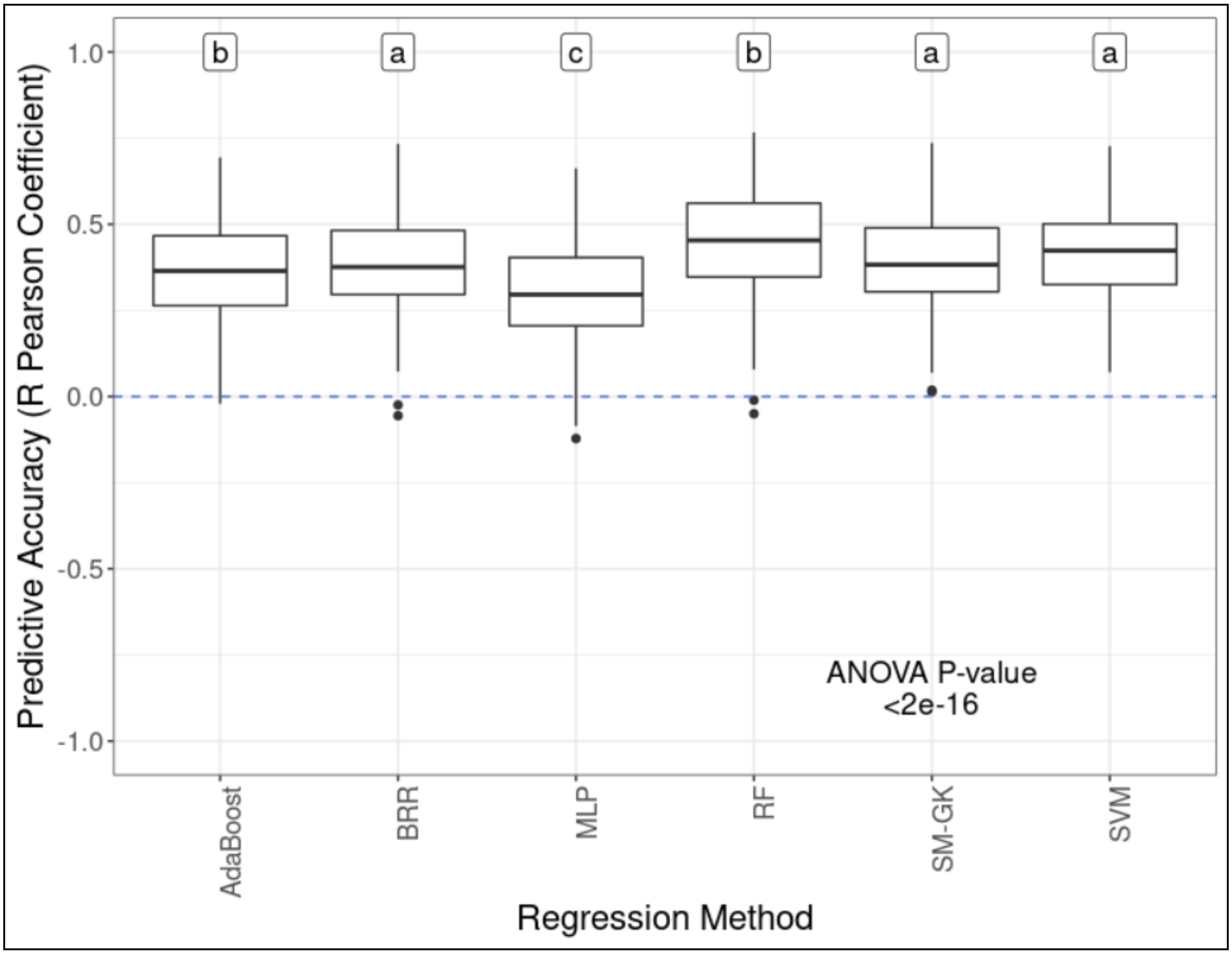

Supplementary Fig. S18. Predictive accuracies for stem circumference BLUP prediction in experimental group 2 (EG2) at site 2 (S2) considering a 4-fold cross validation scheme (50 times repeated), a single-environment model with a nonlinear Gaussian kernel (SM-GK), Bayesian ridge regression (BRR), and several machine learning algorithms for regression (AdaBoost, multilayer perceptron (MLP), random forest (RF), and support vector machine (SVM)). The letters at the top indicate the results from Tukey's multiple comparison test.

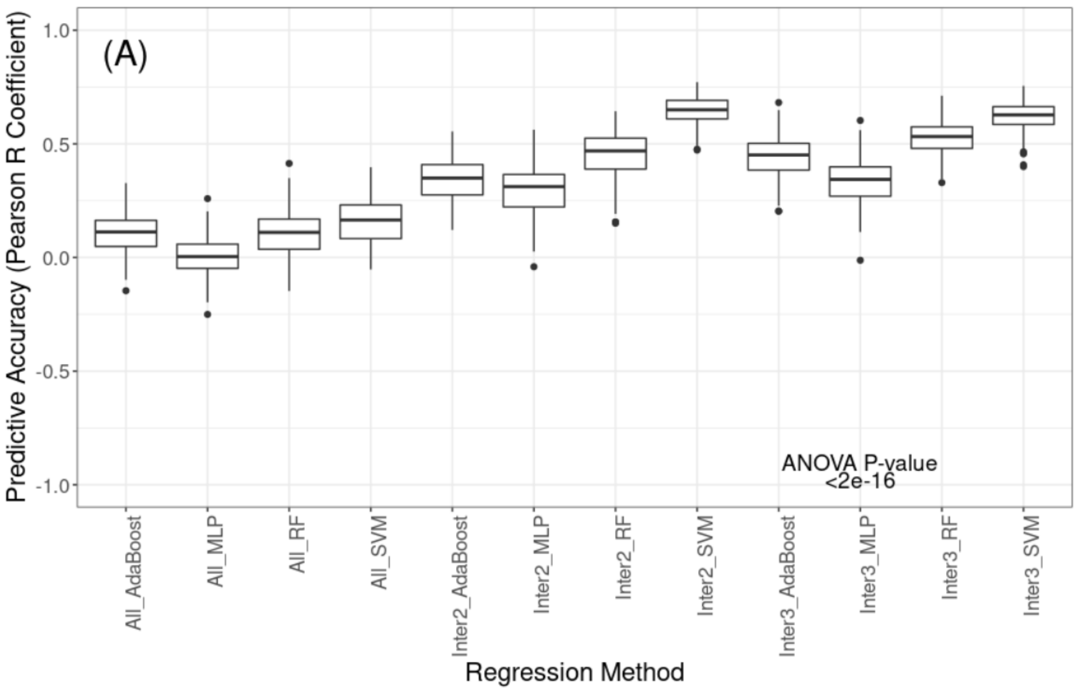

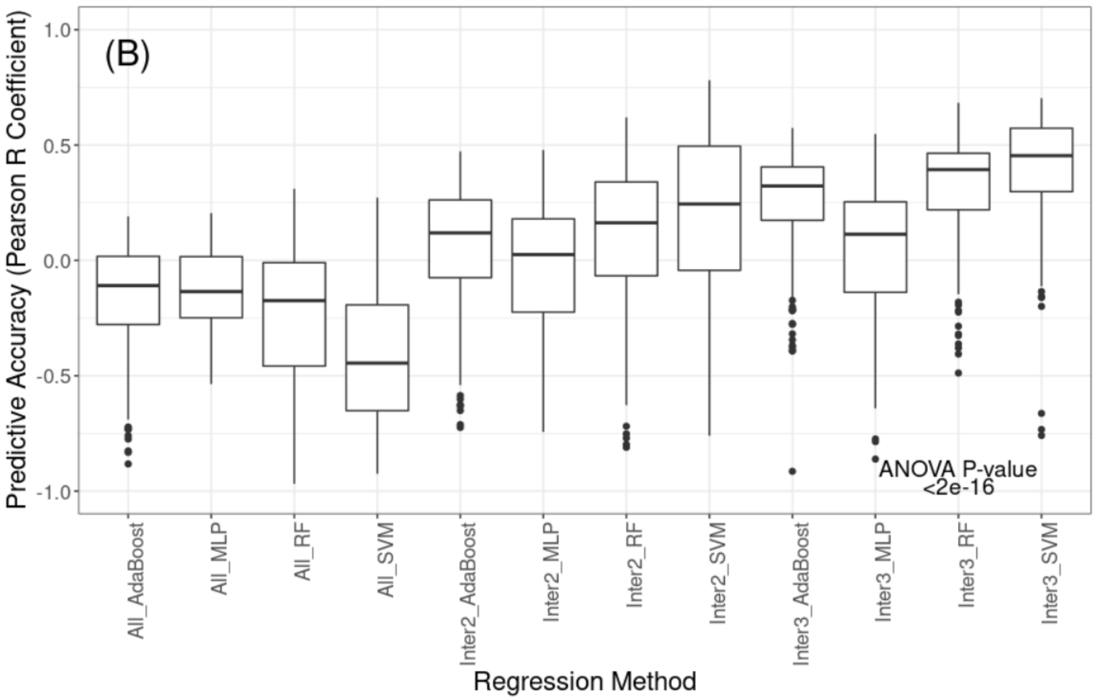

Supplementary Fig. S19. Predictive accuracies for stem circumference BLUP prediction in experimental group 1 (EG1) considering a 4-fold cross validation scheme (repeated 50 times),

different machine learning algorithms for regression (AdaBoost, multilayer perceptron (MLP), random forest (RF), and support vector machine (SVM)), and feature selection (nonincorporation (All), intersection among the three methods established (Inter3), and intersection between at least two out of the three methods established (Inter2)). The letters at the top indicate the results from Tukey's multiple comparison test. In (A), the overall correlation between the predicted and real BLUPs is evaluated, and in (B), the worst correlation is evaluated considering the Pearson correlation analysis performed according to subpopulation structure.

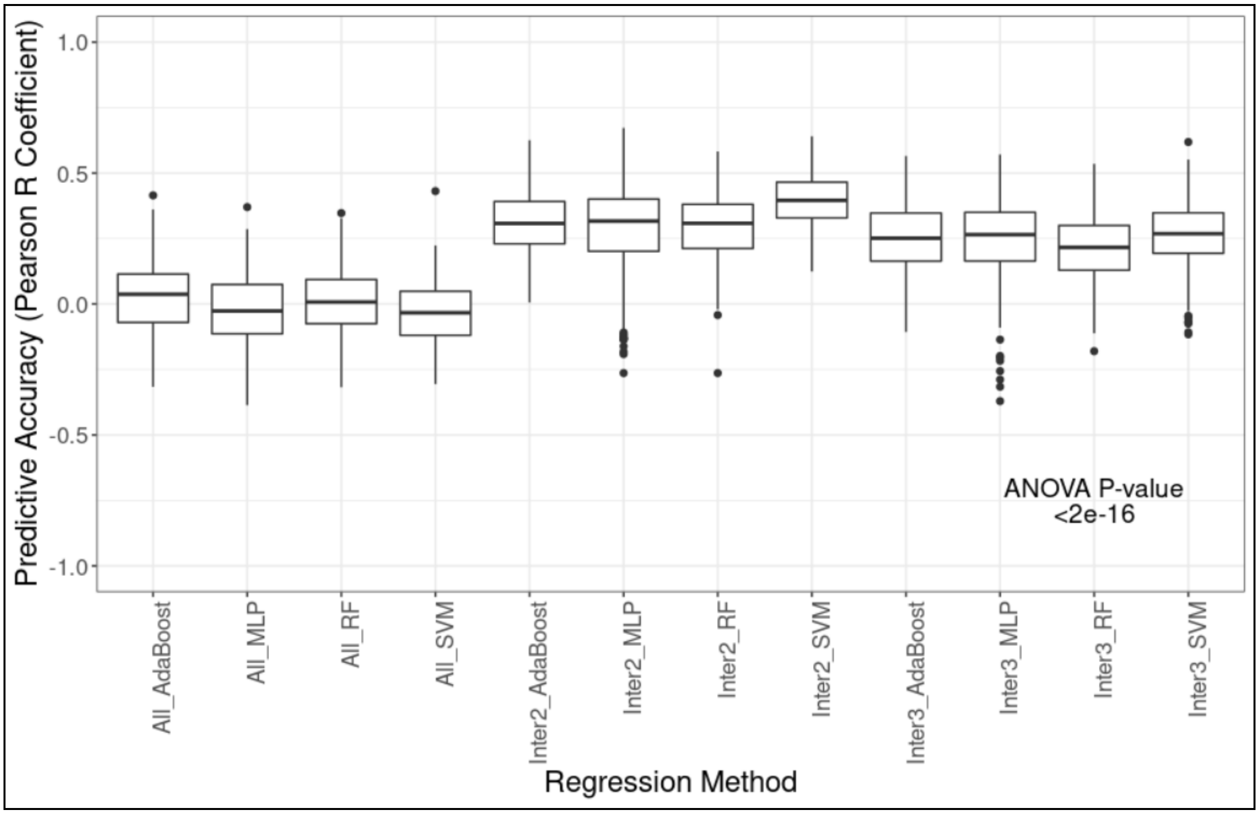

Supplementary Fig. S20. Predictive accuracies for stem circumference BLUP prediction in experimental group 2 (EG2) and the first site (S1) considering a 4-fold cross validation scheme (repeated 50 times), different machine learning algorithms for regression (AdaBoost, multilayer perceptron (MLP), random forest (RF), and support vector machine (SVM)), and feature selection (nonincorporation (All), intersection among the three methods established (Inter3), and intersection between at least two out of the three methods established (Inter2)). The letters at the top indicate the results from Tukey’s multiple comparison test.

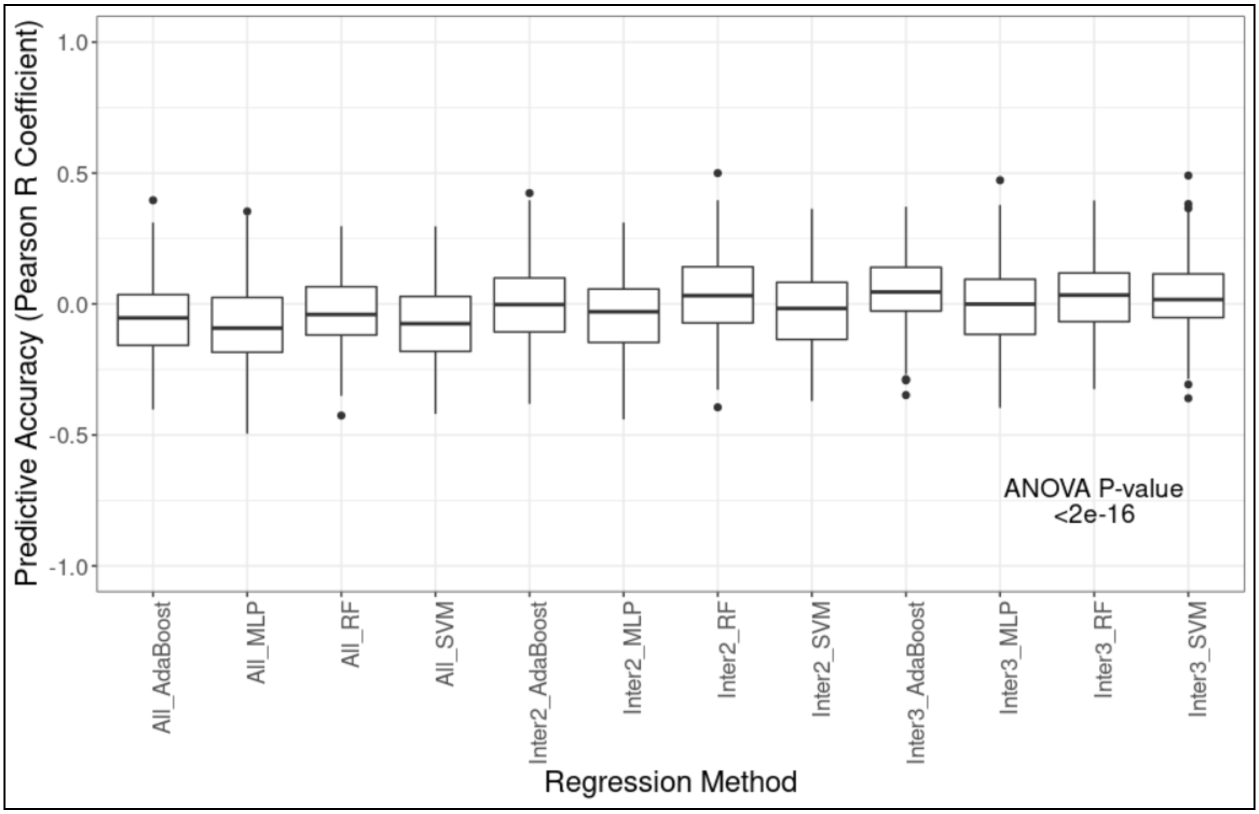

Supplementary Fig. S21. Predictive accuracies for stem circumference BLUP prediction in experimental group 2 (EG2) at the second site (S2) considering a 4-fold cross validation scheme (repeated 50 times), different machine learning algorithms for regression (AdaBoost, multilayer perceptron (MLP), random forest (RF), and support vector machine (SVM)), and feature selection (nonincorporation (All), intersection among the three methods established (Inter3), and intersection among at least two out of the three methods established (Inter2)). The letters at the top indicate the results from Tukey's multiple comparison test.

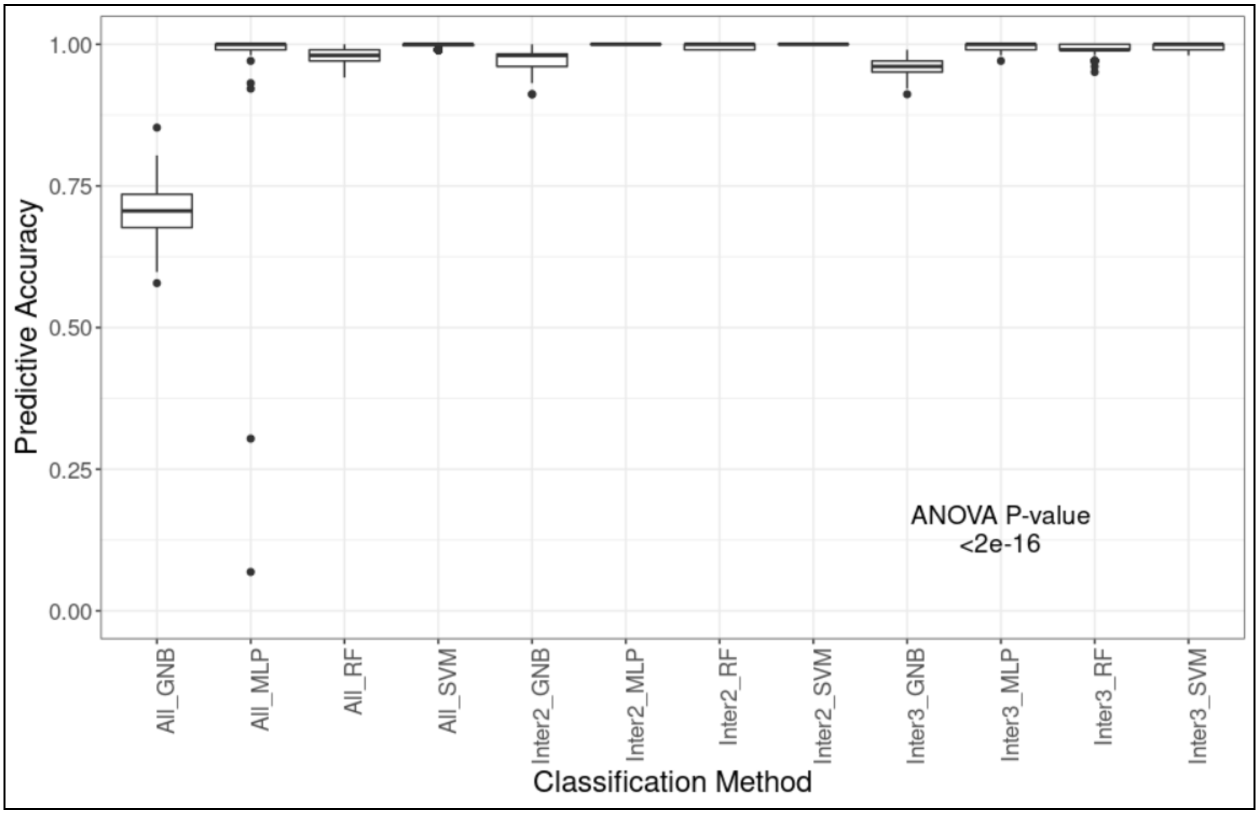

Supplementary Fig. S22. Predictive accuracies for population classification in experimental group 1 (EG1) considering a 4-fold cross validation scheme (repeated 50 times), different classification algorithms for prediction (Gaussian naive Bayes (GNB), multilayer perceptron (MLP), random forest (RF), and support vector machine (SVM)), and feature selection (nonincorporation (All), intersection among the three methods established (Inter3), and intersection between at least two out of the three methods established (Inter2)). The letters at the top indicate the results from Tukey's multiple comparison test.

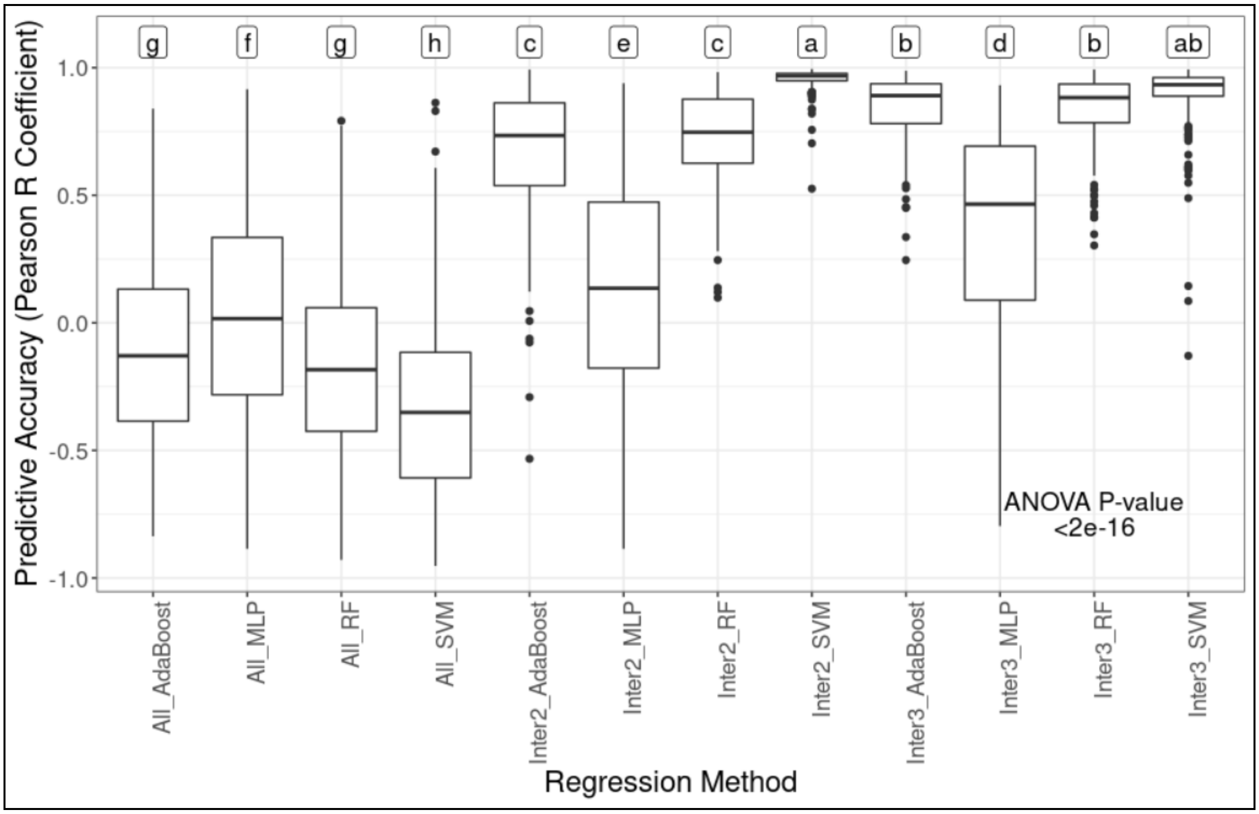

Supplementary Fig. S23. Predictive accuracies for stem circumference BLUP prediction in experimental group 1 (EG1) and the population GT1 x PB235 considering a 4-fold cross validation scheme (repeated 50 times), different machine learning algorithms for regression (AdaBoost, multilayer perceptron (MLP), random forest (RF), and support vector machine (SVM)), and feature selection (nonincorporation (All), intersection among the three methods established (Inter3), and intersection between at least two out of the three methods established (Inter2)). The letters at the top indicate the results from Tukey's multiple comparison test.

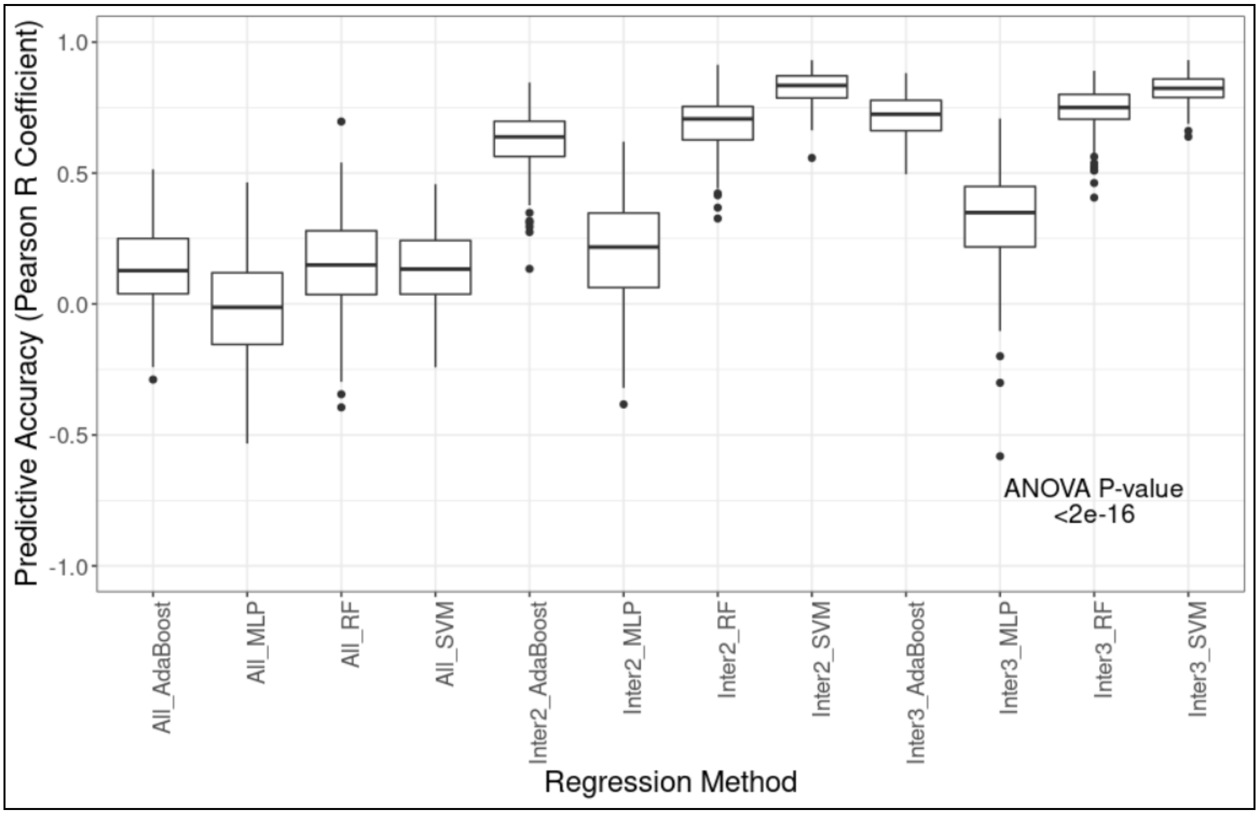

Supplementary Fig. S24. Predictive accuracies for stem circumference BLUP prediction in experimental group 1 (EG1) and the population GT1 x RRIM701 considering a 4-fold cross validation scheme (repeated 50 times), different machine learning algorithms for regression (AdaBoost, multilayer perceptron (MLP), random forest (RF), and support vector machine (SVM)), and feature selection (nonincorporation (All), intersection among the three methods established (Inter3), and intersection between at least two out of the three methods established (Inter2)). The letters at the top indicate the results from Tukey's multiple comparison test.

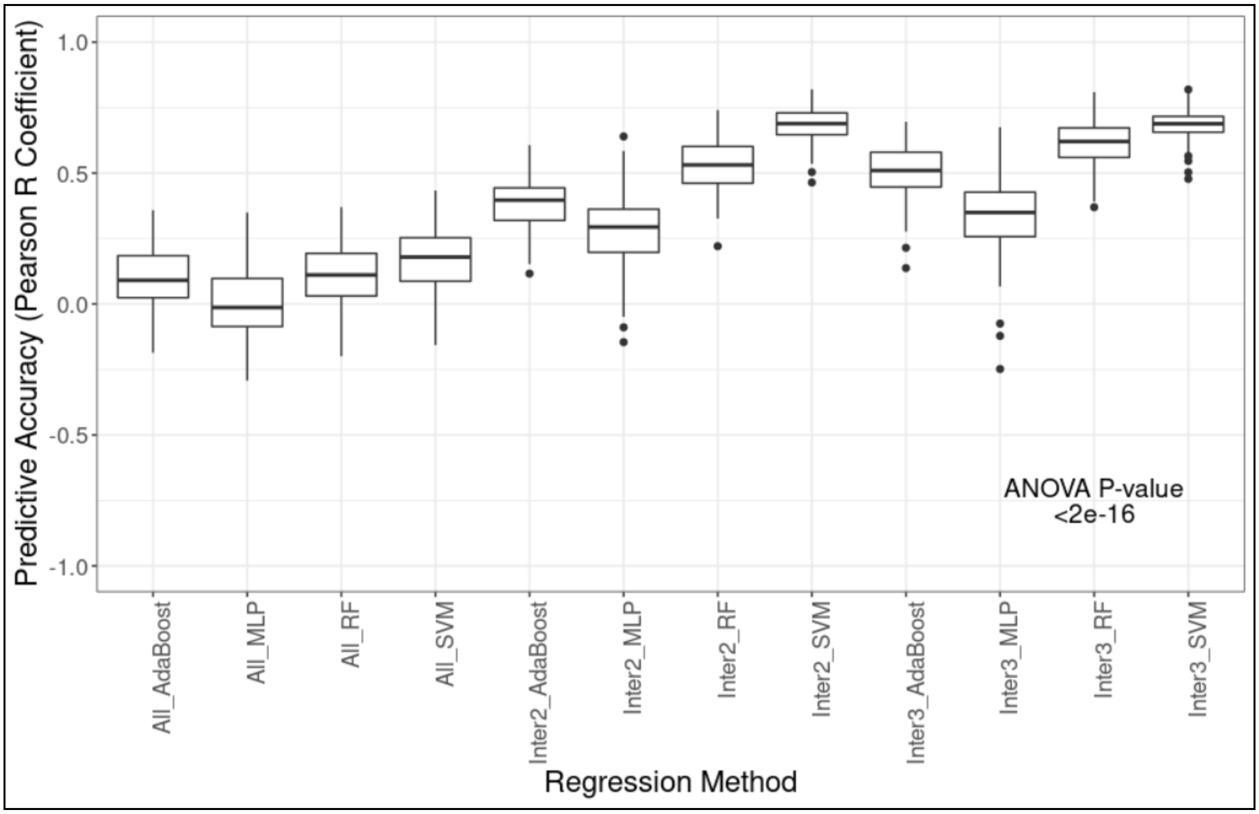

Supplementary Fig. S25. Predictive accuracies for stem circumference BLUP prediction in experimental group 1 (EG1) and the population PR255 x PB217 considering a 4-fold cross validation scheme (repeated 50 times), different machine learning algorithms for regression (AdaBoost, multilayer perceptron (MLP), random forest (RF), and support vector machine (SVM)), and feature selection (nonincorporation (All), intersection among the three methods established (Inter3), and intersection between at least two out of the three methods established (Inter2)). The letters at the top indicate the results from Tukey's multiple comparison test.

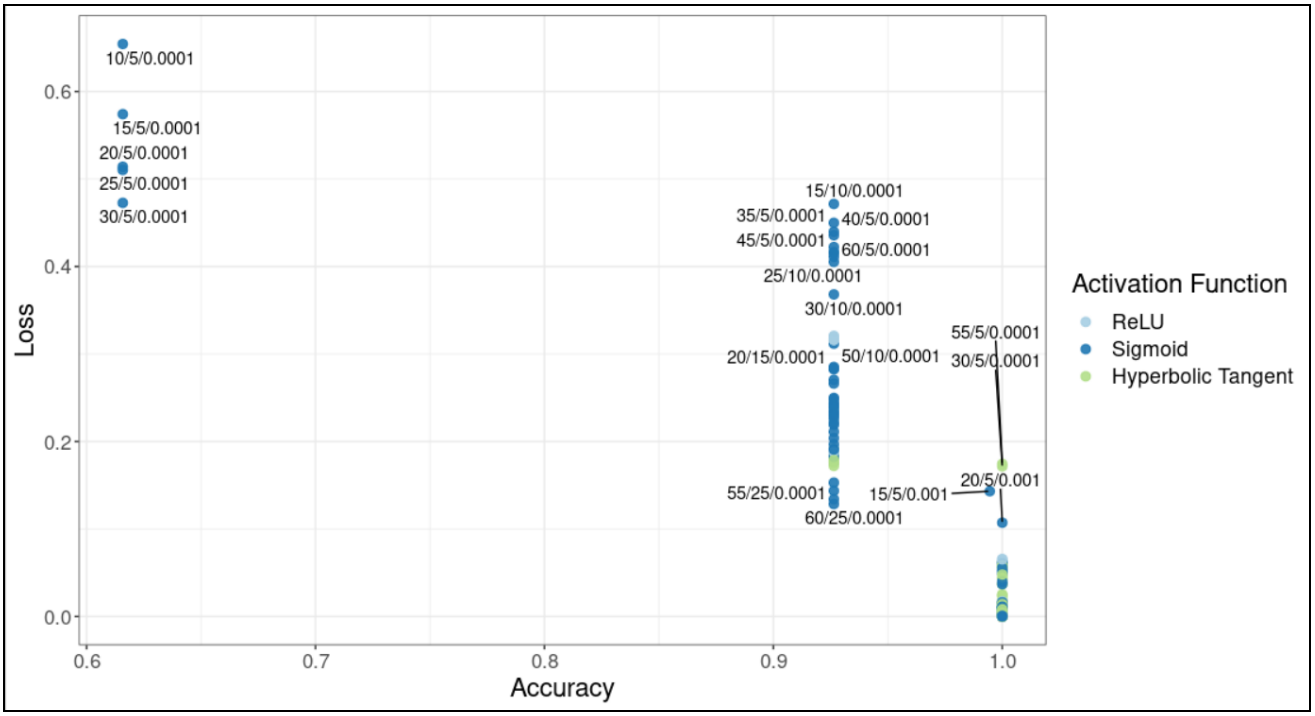

Supplementary Fig. S26. Hyperparameter search for subpopulation prediction in experimental group 1 (EG1). Each point represents a different combination of hyperparameters, considering (a) neurons in the first hidden layer, (b) neurons in the second hidden layer, (c) the learning rate, and (d) the activation function. These combinations were evaluated in the development set considering accuracy and the loss function evaluations.

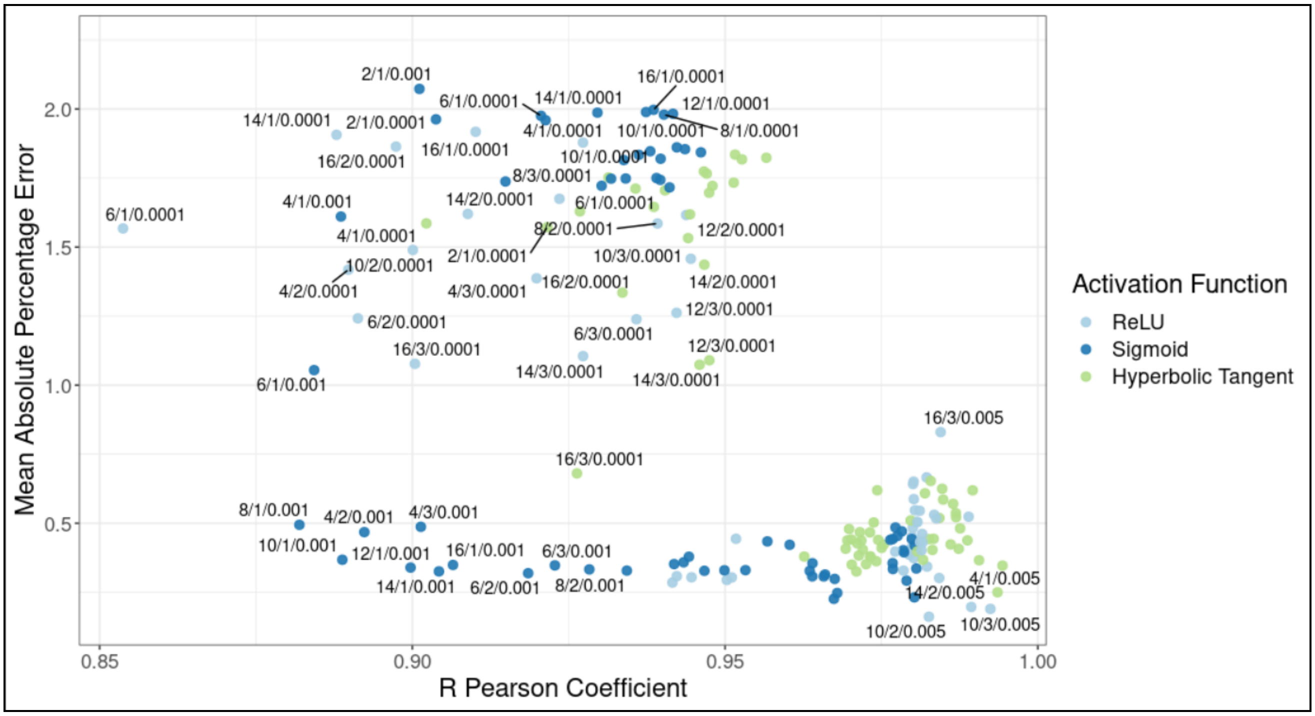

Supplementary Fig. S27. Hyperparameter search for stem circumference BLUP prediction in experimental group 1 (EG1) for the population GT1 x PB235. Each point represents a different combination of hyperparameters, considering (a) neurons in the first hidden layer, (b) neurons in the second hidden layer, (c) the learning rate, and (d) the activation function. These combinations were evaluated in the development set considering the mean absolute percentage error (MAPE) and the R Pearson coefficient.

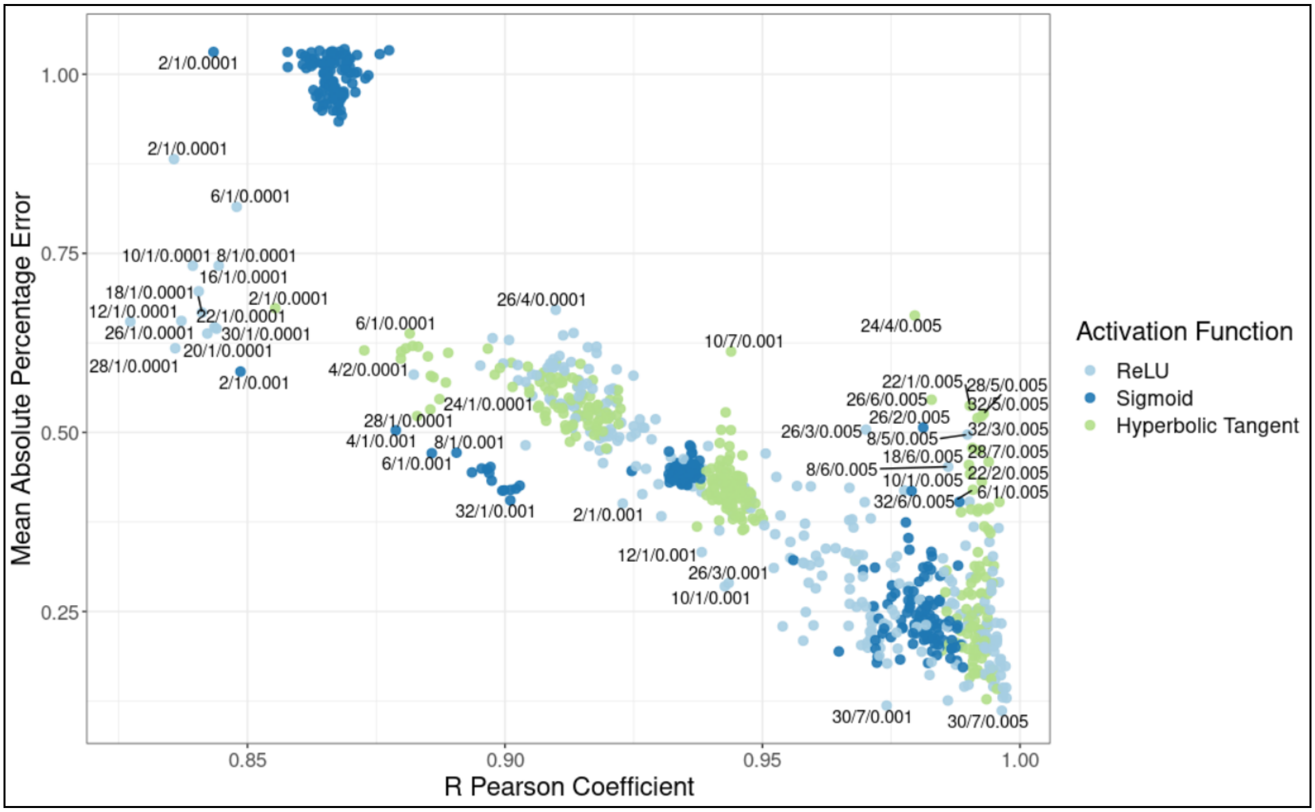

Supplementary Fig. S28. Hyperparameter search for stem circumference BLUP prediction in experimental group 1 (EG1) for the population GT1 x RRIM701. Each point represents a different combination of hyperparameters, considering (a) neurons in the first hidden layer, (b) neurons in the second hidden layer, (c) the learning rate, and (d) the activation function. These combinations were evaluated in the development set considering the mean absolute percentage error (MAPE) and the R Pearson coefficient.

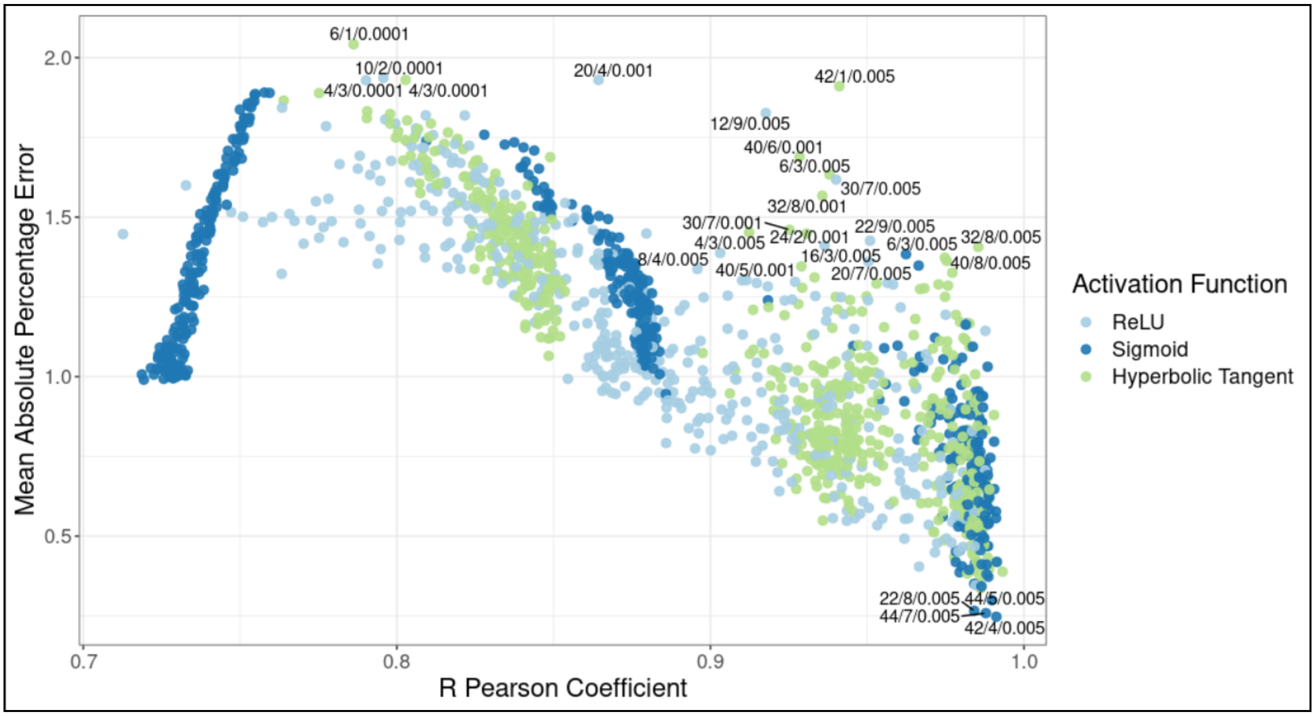

Supplementary Fig. S29. Hyperparameter search for stem circumference BLUP prediction in experimental group 1 (EG1) for the population PR255 x PB217. Each point represents a different combination of hyperparameters, considering (a) neurons in the first hidden layer, (b) neurons in the second hidden layer, (c) the learning rate, and (d) the activation function. These combinations were evaluated in the development set considering the mean absolute percentage error (MAPE) and the R Pearson coefficient.

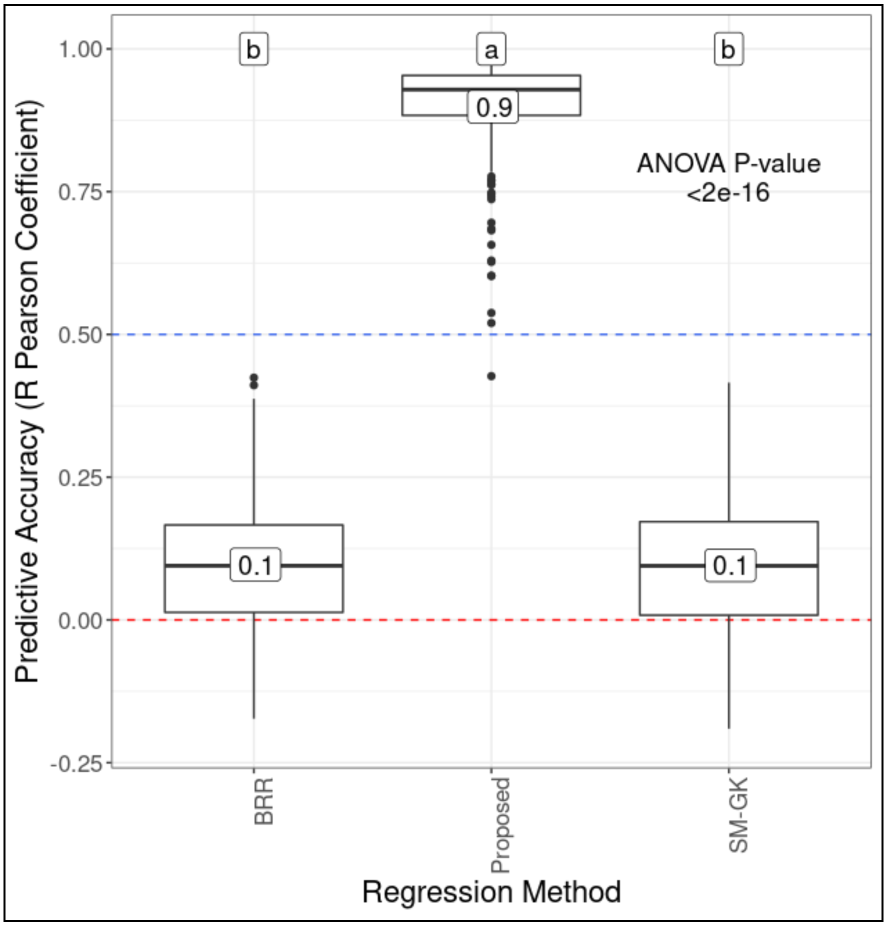

Supplementary Fig. S30. Predictive accuracies for stem circumference BLUP prediction in experimental group 1 (EG1) and the population GT1 x PB235 considering a 4-fold cross validation scheme (repeated 50 times), the proposed approach and traditional genomic prediction methods (a single-environment model with a nonlinear Gaussian kernel (SM-GK) and Bayesian ridge regression (BRR)). The letters at the top indicate the results from Tukey's multiple comparison test.

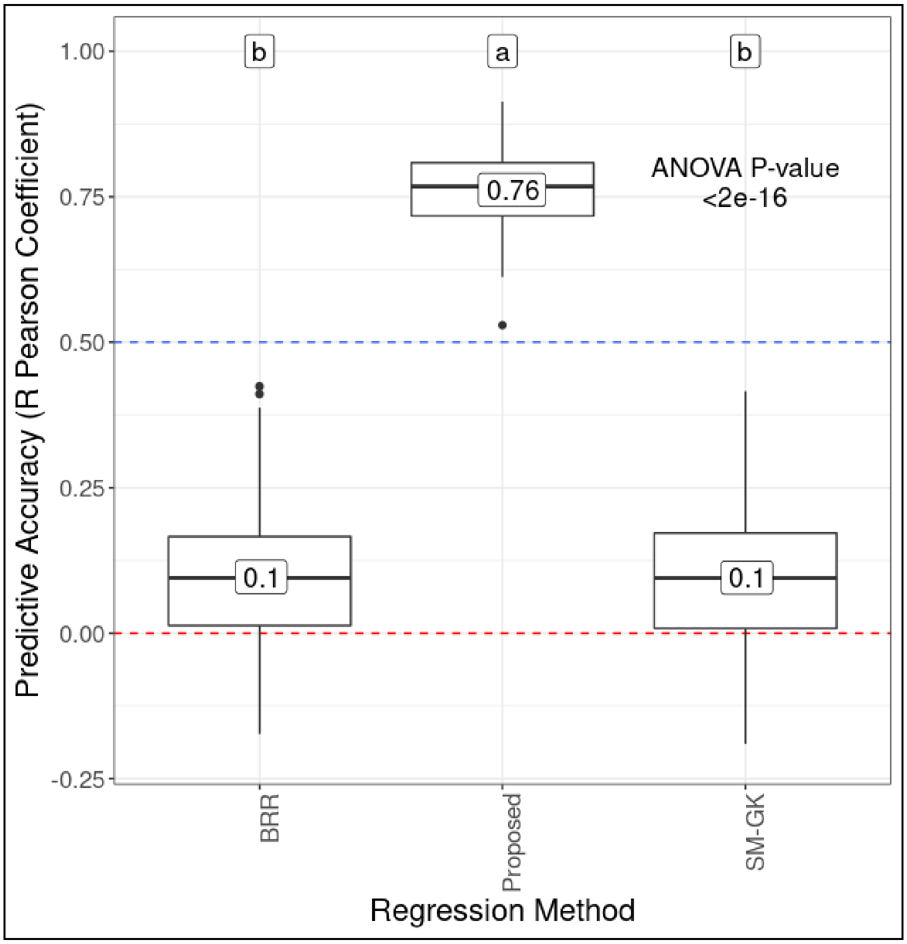

Supplementary Fig. S31. Predictive accuracies for stem circumference BLUP prediction in experimental group 1 (EG1) and the population GT1 x RRIM701 considering a 4-fold cross validation scheme (repeated 50 times), the proposed approach and traditional genomic prediction methods (a single-environment model with a nonlinear Gaussian kernel (SM-GK) and Bayesian ridge regression (BRR)). The letters at the top indicate the results from Tukey's multiple comparison test.

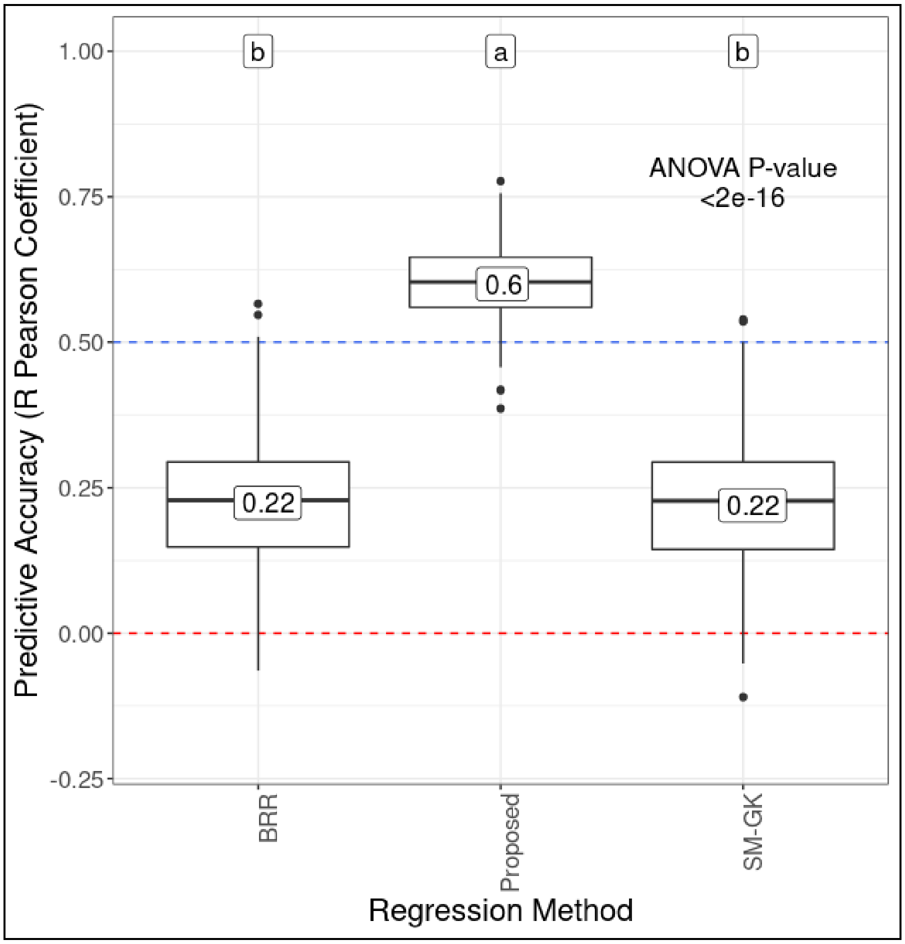

Supplementary Fig. S32. Predictive accuracies for stem circumference BLUP prediction in experimental group 1 (EG1) and the population PR255 x PB217 considering a 4-fold cross validation scheme (repeated 50 times), the proposed approach and traditional genomic prediction methods (a single-environment model with a nonlinear Gaussian kernel (SM-GK) and Bayesian ridge regression (BRR)). The letters at the top indicate the results from Tukey's multiple comparison test.
